# Irreversible light-activated SpyLigation mediates split-protein assembly in 4D

**DOI:** 10.1038/s41596-023-00938-0

**Published:** 2024-01-22

**Authors:** Brizzia G. Munoz-Robles, Cole A. DeForest

**Affiliations:** 1 Department of Bioengineering, University of Washington, Seattle, WA, USA.; 2 Institute of Stem Cell & Regenerative Medicine, University of Washington, Seattle, WA, USA.; 3 Department of Chemical Engineering, University of Washington, Seattle, WA, USA.; 4 Department of Chemistry, University of Washington, Seattle, WA, USA.; 5 Molecular Engineering & Sciences Institute, University of Washington, Seattle, WA, USA.; 6 Institute for Protein Design, University of Washington, Seattle, WA, USA.

## Abstract

The conditional assembly of split-protein pairs to modulate biological activity is commonly achieved by fusing split-protein fragments to dimerizing components that bring inactive pairs into close proximity in response to an exogenous trigger. However, current methods lack full spatial and temporal control over reconstitution, require sustained activation and lack specificity. Here light-activated SpyLigation (LASL), based on the photoregulation of the covalent SpyTag (ST)/SpyCatcher (SC) peptide–protein reaction, assembles nonfunctional split fragment pairs rapidly and irreversibly in solution, in engineered biomaterials and intracellularly. LASL introduces an *ortho*-nitrobenzyl(*o*NB)-caged lysine into SC’s reactive site to generate a photoactivatable SC (pSC). Split-protein pairs of interest fused to pSC and ST are conditionally assembled via near-ultraviolet or pulsed near-infrared irradiation, as the uncaged SC can react with ST to ligate appended fragments. We describe procedures for the efficient synthesis of the photocaged amino acid that is incorporated within pSC (<5 days) as well as the design and cloning of LASL plasmids (1–4 days) for recombinant protein expression in either *Escherichia coli* (5–6 days) or mammalian cells (4–6 days), which require some prior expertise in protein engineering. We provide a chemoenzymatic scheme for appending bioorthogonal reactive handles onto *E. coli*-purified pSC protein (<4 days) that permits LASL component incorporation and patterned protein activation within many common biomaterial platforms. Given that LASL is irreversible, the photolithographic patterning procedures are fast and do not require sustained light exposure. Overall, LASL can be used to interrogate and modulate cell signaling in various settings.

## Introduction

**Fig. 1 |:**
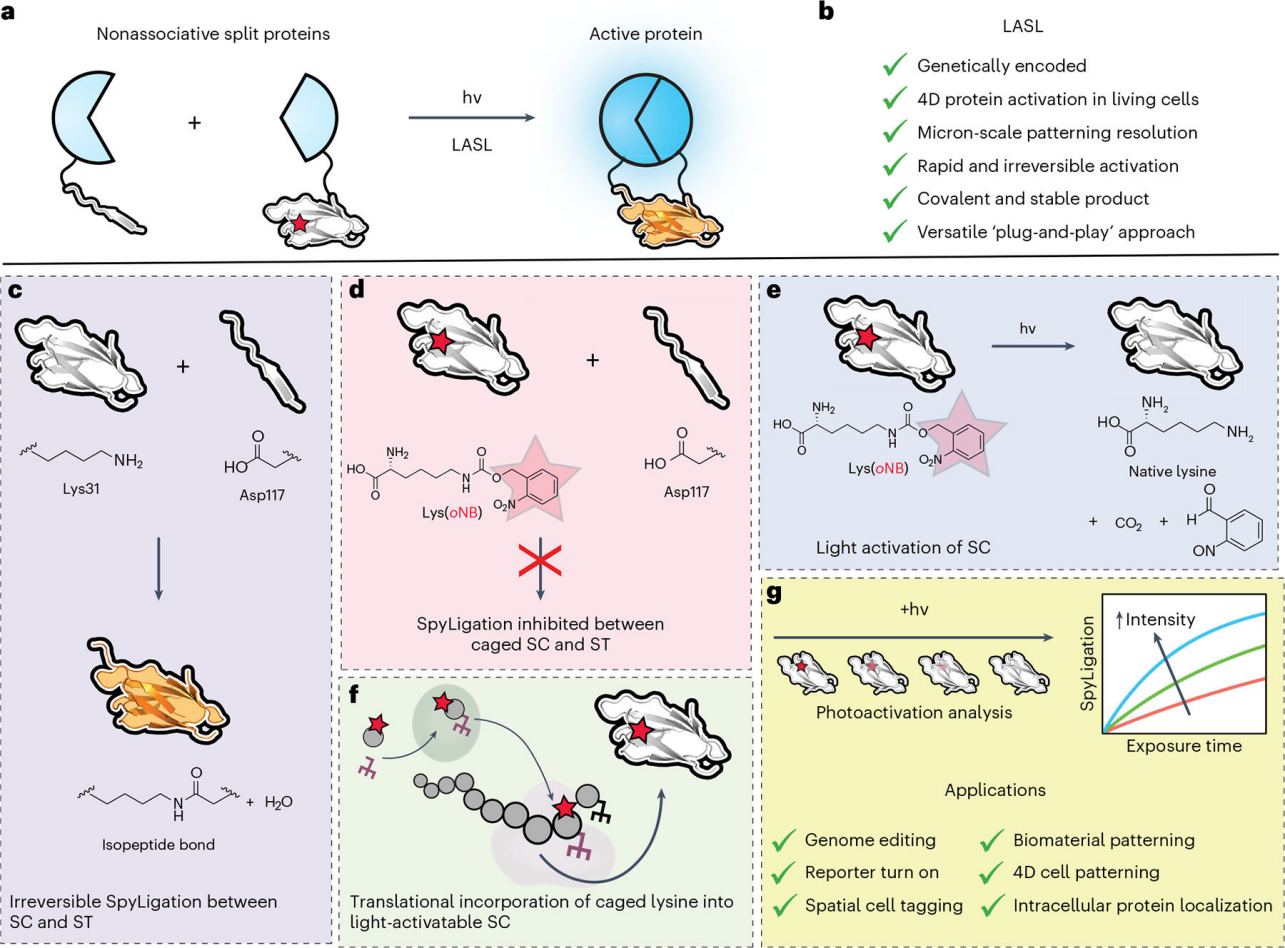
LASL affords complete spatiotemporal control over protein activation. **a**, When pSC and ST are genetically fused to otherwise nonassociative split proteins, irreversible protein assembly and functional activation is chemically regulated with light (hν, *λ* = 365 nm or 740 nm). **b**, The use of LASL to irreversibly activate split proteins offers many distinct advantages over existing optogenetic strategies and can be used to interrogate a variety of biological functions in 4D. **c**, Irreversible SpyLigation of the SC/ST pair occurs between SC’s Lys31 and ST’s Asp117 residues. **d**, Owing to the bulky photocage masking its reactive amine, pSC remains inactive and unable to interact with or covalently bind ST. **e**, With user-directed light exposure, the critical lysine is liberated to generate newly uncaged SC, which is capable of spontaneous isopeptide bond formation with ST. **f**, A photocaged lysine is translationally incorporated at the active site of SC via an unnatural tRNA/tRNA synthetase pair, giving pSC. **g**, SpyLigation can be controlled in a dosage-dependent manner. Figure adapted from ref. 11, Springer Nature Ltd.

Conditional split-protein assembly has emerged as a powerful tool for interrogating cell signaling and guiding biological fate. In this strategy, proteins are genetically split into biologically inactive fragments that can be proximally reconstituted into functional species. Triggered reconstitution is commonly achieved by genetically fusing the split pairs with inducible dimerizing partner domains, whereby an external factor brings the partner pair into close physical proximity^1^. Such split-protein reassembly has been used in a variety of applications including probing protein–protein interactions^2^, investigating/stimulating protein signaling pathways^3^ and genome editing^4,5^. Despite its utility, more widespread application of split-protein systems has been largely limited by the dimerization chemistries. For example, small molecule-dependent protein fragment reconstitution (e.g., coumermycin homodimerization of GyrB^6^, rapamycin-induced heterodimerization of FKBP/FRB2 (ref. 7)) cannot be readily regulated with full spatial and/or temporal control. Furthermore, although spatiotemporal direction over protein reconstitution has been achieved in systems using optogenetic dimerizing protein pairs that bind open light irradiation (e.g., magnets^8^, PhyB–PIF^9^, Cry2/CIB1 (ref. 10)), the interactions are typically reversible, afford only binary specification and cannot readily be governed in full three-dimensional (3D) space. To address these limitations, we recently developed a genetically encoded and light-activated SpyLigation (LASL) technique to rapidly and irreversibly reassemble nonfunctional split fragment pairs with four-dimensional (4D) control in a dose-dependent manner using cytocompatible light^11^ (Fig. 1). Here, we discuss how to design and apply LASL to photoligate and functionally reconstitute split-protein pairs in solution, throughout biomaterials and within living mammalian cells.

### Development of the protocol

LASL’s design is inspired by the rationally engineered split-protein pair, SpyCatcher (SC, 113 amino acids, 12.1 kDa) and SpyTag (ST, 13 amino acids, 1.5 kDa) that undergoes spontaneous covalent coupling through isopeptide bond formation between SC’s critical lysine (i.e., Lys(31)) and an essential aspartic acid on ST^12^. As developed by the Howarth laboratory, the SpyLigation reaction is highly efficient, occurs in minutes and is robust under diverse conditions of pH, temperature and buffer. Over the past decade, ST and SC have been genetically fused to over 500 proteins for applications in cell surface localization, increasing protein stability, protein assembly into macromolecular structures, biochemical decoration of hydrogels, and even for stabilizing and improving split-protein complementation^13,14^. However, while SpyLigation has proven a powerful tool for a wide variety of applications, its intrinsic spontaneity has precluded its usage in scenarios necessitating spatial and/or temporal specification.

Photoresponsive chemistries have emerged as a popular tool for controlling various biological processes, more so as advances in photolithographic patterning techniques allow light to be directed in user-specified 3D locations and times, at micrometer-scale resolution^15^ in a noninvasive manner (Box 1). Thus, to gain high spatiotemporal control over the spontaneous SpyLigation reaction, we installed a photocaged lysine analog, *N*ε-(*o*-nitrobenzyloxycarbonyl)-l-lysine (Lys(*o*NB)), at SC’s critical lysine to generate a photoactivatable SC (pSC). The photolabile protecting group (i.e., *o*NB photocage) renders pSC inactive until cytocompatible near-ultraviolet (UV) (*λ* = 365 nm)^16^ or two-photon (*λ* = 740 nm) light exposure, which cleaves the caging group and restores function^11^. Site-specific insertion of the noncanonical amino acid (ncAA) was accomplished using genetic code expansion techniques^17^, whereby an orthogonal aminoacyl–transfer (t)RNA synthetase (aaRS)/tRNA pair engineered for the efficient incorporation of Lys(*o*NB) during protein translation inserts the photocaged lysine at an amber stop codon substituted at the Lys(31) of SC. Translational installation of the Lys(*o*NB) can be accomplished in both bacterial and mammalian systems by using an aaRS/tRNA pair orthogonal to the endogenous tRNAs in the expression host. For bacterial expression of pSC, we designed an aaRS/tRNA plasmid encoding two copies of a mutant *Methanosarcina maize* pyrrolysyl–tRNA synthetase (*Mm*PylRS; Y306M, L309A, C348A, Y384F) (ref. 18), which has been previously utilized for Lys(*o*NB) incorporation and one copy of the Pyl–tRNA_CUA_ within the pEVOL^19^ vector (pEVOL–NBK-1). To introduce Lys(*o*NB) site specifically in mammalian cells, we used a plasmid encoding a mutant *Methanosarcina bakeri* synthetase (*Mb*PylRS; Y271A, Y349F) previously applied to incorporate various noncanonical lysine species^20^—but not with Lys(*o*NB)—and four copies of an engineered M15–tRNA_CUA_, which has been used in pyrrolysine-based systems to increase incorporation of noncanonical amino acids (pNeu–hMbPylRS–4xU6M15) (ref. 21).

We established and characterized LASL photokinetics by recombinantly expressing pSC, wild-type (WT) SC, and glutathione *S*-transferase SpyTag fusion (GST–ST) proteins in *Escherichia coli*. Purified proteins were characterized and identified by sodium dodecyl sulfate–polyacylamide gel electrophoresis (SDS–PAGE) and liquid chromatography–mass spectrometry (LC–MS). Upon light exposure, an observed mass shift corresponding to photocleavage of pSC’s *o*NB cage was observed, confirming its proper incorporation. Through an SDS–PAGE gel shift analysis of purified pSC protein reacted with GST–ST, a band corresponding to the SpyLigated product was only observed in light-exposed solutions, demonstrating that pSC functionality could be controlled and restored through photoactivation. The kinetic constant for pSC activation was determined by exposing pSC to various amounts of light and quantifying the disappearance of the starting proteins and appearance of the SpyLigated product. Analysis revealed a first-order uncaging constant of ~0.07 cm^2^ J^−1^ and a half-life constant of 9 J cm^−2^. To assess reaction specificity in complex environments, LASL was performed in bacterial and mammalian lysate in which only the expected SpyLigation reaction was observed. Collectively, experiments established that LASL proceeded specifically and could be controlled in a light dose-dependent manner.

In the context of controlled protein reassembly, LASL is a versatile plug-and-play approach in which the pSC/ST pair can be genetically fused to split-protein fragments for conditional protein reconstitution and activation. So far, we have exploited LASL’s robust-yet-specific chemistry to functionally reassemble split UnaG fluorescent proteins, NanoLuc luciferase enzymes and Cre recombinase genome editors in various settings, including in solution, throughout hydrogel biomaterials and within living cells in full 4D space via both single- and multiphoton-based processes.

### Overview of the procedure

This protocol consists of five procedures that include the design and generation of components necessary for LASL and its application in solution, biomaterials and mammalian cells. Procedure 1 describes an improved three-step synthetic route for generation of the Lys(*o*NB) ncAA that is required for pSC expression. Procedure 2 describes the design of LASL split proteins including identifying optimal fusion arrangements of ST/SC, plasmid vector design based on expression in bacterial or mammalian cells, and molecular cloning steps to insert the amber stop codon at the critical lysine of SC. Procedure 3 describes the recombinant protein expression of *E. coli*-based constructs using genetic code expansion and testing of the LASL components in solution. Procedure 4 describes a sortase-mediated reaction to site-specifically append bioorthogonal reactive handles on pSC for its incorporation and LASL patterning in biomaterials. Procedure 5 describes a mammalian cell transfection procedure for intracellular protein localization/activation via LASL. Procedures 3–5 include photolithographic patterning procedures specific to each setting.

### Application of the method

#### LASL in solution

**Fig. 2 |:**
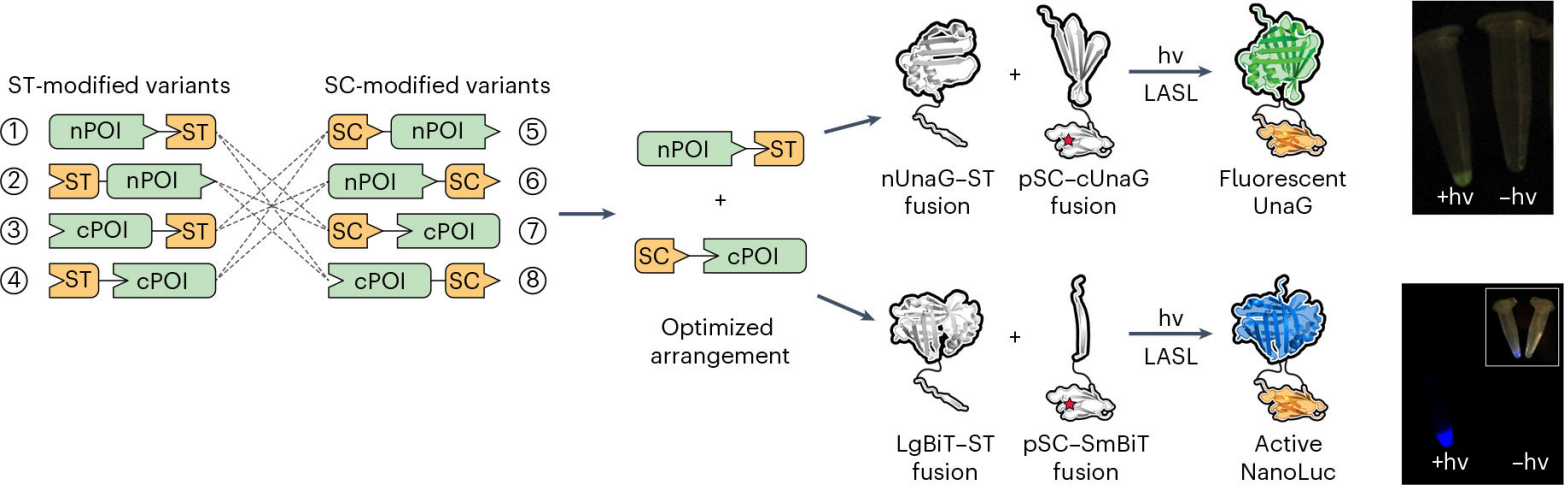
In-solution assembly of UnaG and NanoLuc through LASL of split-protein fragments. The eight possible variants of ST/SC fused to the N or C terminus of the split POI fragments were cloned and recombinantly expressed in *E. coli* to screen for combinations that exhibited maximum split-protein reconstitution and activity. The optimized arrangement nPOI–ST and SC–cPOI was used to generate a LASL-assembled fluorescent UnaG and bioluminescent NanoLuc split-protein system. Figure adapted from ref. 11, Springer Nature Ltd.

Using a previously validated split site for a fluorescent UnaG protein^22^, we cloned all possible fusion arrangements in bacterial expression vectors where the split UnaG fragments were functionalized with ST or WT SC at the N or C terminus (Fig. 2). We recombinantly expressed the fusions in *E. coli* and selected the brightest split-protein combination (nUnaG–ST + pSC–cUnaG) for further LASL experiments. A simple cloning procedure was used to substitute SC’s critical Lys(31) with the TAG codon required for pSC protein expression. Purified pSC and ST UnaG protein pairs displayed minimal fluorescence when unexposed to light but gained fluorescence in a light dose-dependent manner following LASL-mediated assembly. To highlight the versatility of LASL, the same N- and C-terminal split-protein fragment arrangement used for UnaG (nPOI–ST + pSC–cPOI, where POI refers to the protein of interest) was successfully translated to reconstitute a split NanoLuc bioluminescent enzyme protein^23^, demonstrating the generalizability of the strategy.

#### LASL in biomaterials

**Fig. 3 |:**
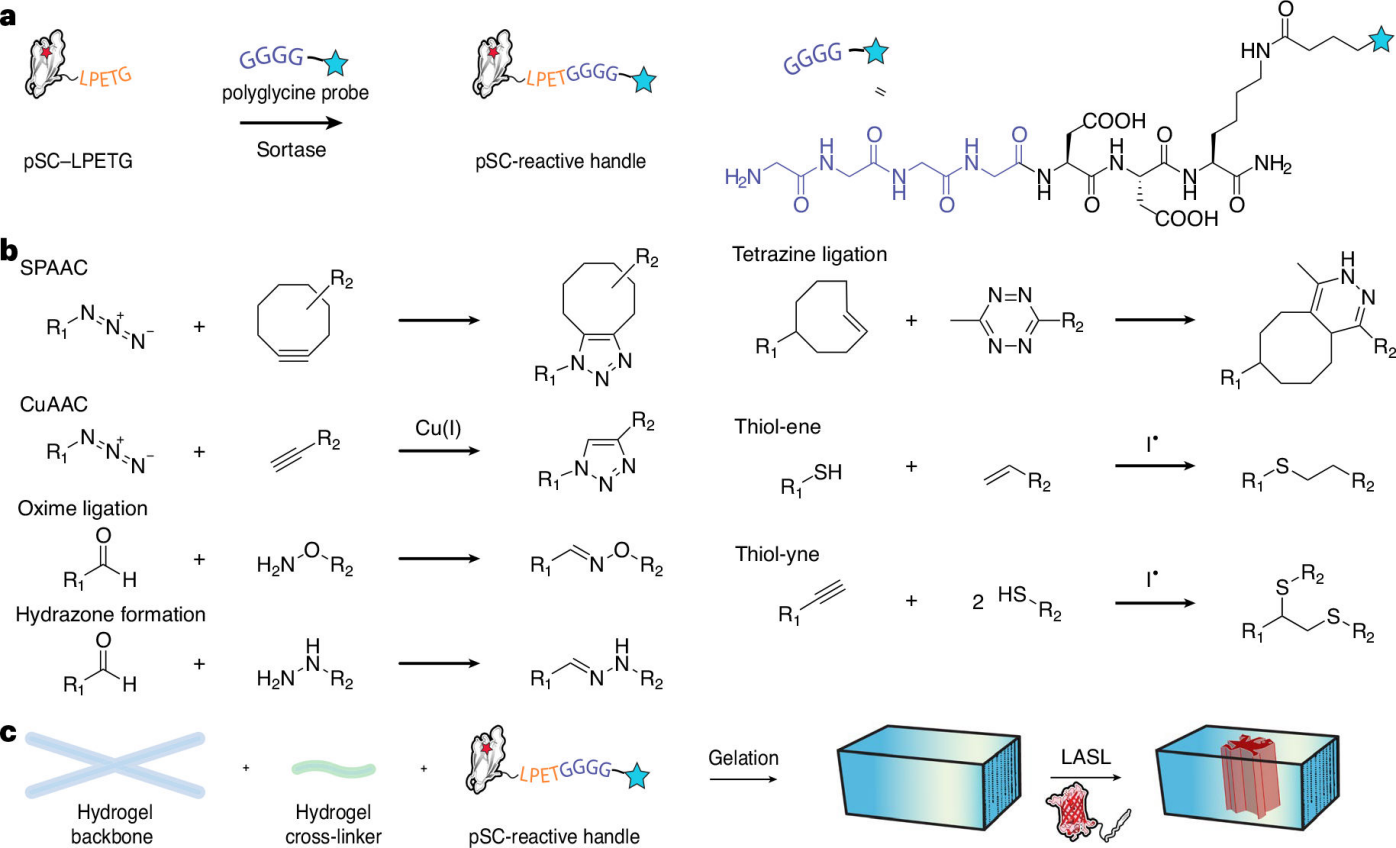
LASL enables site-specific patterned protein localization and split-protein assembly in biomaterials. **a,** The pSC protein is appended with a genetically encoded sorting signal (LPETG), for sortagging with a polyglycine probe containing a reactive handle for biomaterial incorporation. **b,** Common chemistries that can be utilized to incorporate LASL in a variety of biomaterials. R_1_ and R_2_ can each refer to the pSC protein or biomaterial components. The reactive handle on the pSC fragment is defined by the functional group conjugated to the polyglycine probe utilized during sortagging of pSC. **c**, General uniform incorporation of the chemoenzymatically functionalized pSC into a hydrogel of interest. Photoactivation permits spatiotemporally defined immobilization of POI–SpyTag constructs via LASL. **a** and c adapted from ref. 11, Springer Nature Ltd. **b** reprinted with permission from ref. 58, Elsevier.

Controlling protein activity in biomaterials is important for engineering cell culture platforms that recapitulate essential features of native tissue. Although photochemical techniques have been successfully exploited to immobilize full-length proteins within polymeric hydrogels, genetically encoded photochemistries to control protein tethering have not yet been established. Furthermore, during the photopatterning process of full-length proteins, cells embedded in the biomaterial are flooded with active protein in a nonspecific manner, which undermines efforts to spatiotemporally direct cell fate. LASL addresses these limitations and has been successfully exploited to pattern protein immobilization and split-protein reassembly within hydrogels. Genetically encoding a carboxy C-terminal sortase amino acid recognition motif (e.g., LPETG) on the pSC variant (pSC–LPETG), allows the chemoenzymatic modification of pSC with polyglycine peptide probes containing a reactive handle via sortase-based transpeptidation^24–28^ (Fig. 3a). Since various reactive handles can be conjugated to the polyglycine probe, monotagged pSC can be incorporated into hydrogels formed through almost any gelation chemistry (e.g., click reactions, (meth)acrylate, thiol-ene, Michael-type, inverse electron demand Diels–Alder) (Fig. 3b,c). We sortagged pSC with an azide-containing probe such that it could be readily immobilized within poly(ethylene glycol) (PEG)-based hydrogels via a strain-promoted azide-alkyne cycloaddition (SPAAC) reaction^29–32^. pSC-decorated gels were patterned by near-UV or two-photon stimulation (with the addition of a photosensitizer), where the *o*NB cage was cleaved in light-exposed regions, converting pSC to its active form and permitting localized immobilization of SpyTagged proteins via LASL. The spatial patterning of a fluorescent SpyTagged protein and split-protein reconstitution of UnaG in hydrogels was demonstrated.

#### LASL in mammalian cells

**Fig. 4 |:**
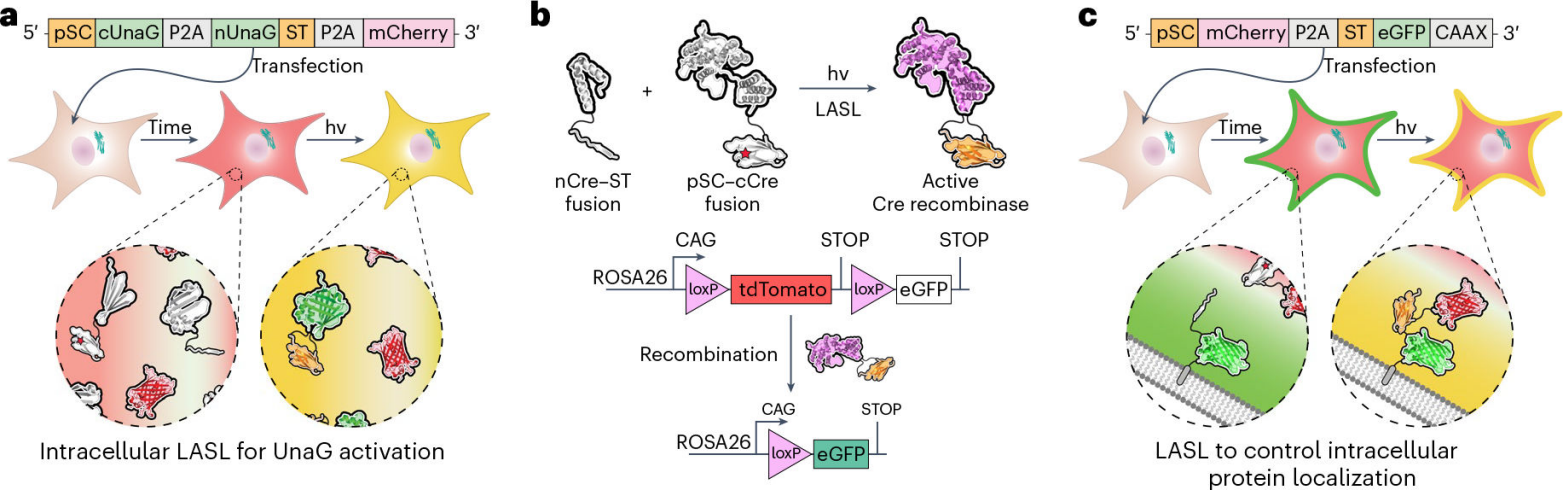
LASL-mediated split-protein activation in mammalian cells. **a,** A schematic of the pSC–UnaG gene cassette used to prime cells for LASL of nonassociative UnaG fragments. **b**, Cre recombinase split into inactive N- (nCre) and C-terminal (cCre) fragments and respectively genetically fused to ST and pSC can be functionally reassembled using LASL. **c**, A schematic of the gene cassette used to prime cells for LASL-mediated plasma membrane labeling, where a CAAX-anchored enhanced green fluorescent protein (eGFP) fused with ST was covalently ligated with cytosolic pSC–mCherry upon light exposure. Figure adapted from from ref. 11, Springer Nature Ltd.

Regulating protein activity in mammalian cells is critical for interrogating cell signaling and guiding cell function. As both reactive components in LASL are genetically encoded, LASL has been successfully exploited to pattern irreversible protein localization as well as covalent protein reassembly and concomitant activation intracellularly. The two split-protein fragments genetically fused with either ST or pSC were cloned into a single mammalian expression vector, whereby the LASL components were separated by a self-cleaving P2A peptide sequence^33^. Using a single polycistronic construct has several advantages as it helps ensure near equimolar expression levels of each fragment. A fluorescent protein preceded by a P2A sequence can also be appended to the end of the construct to provide an internal standard to account for transfection variations. Localization sequences, such as the plasma membrane-localizing CAAX motif from K-Ras^34^ or nuclear localization sequence, can also be genetically incorporated. We specifically cotransfected LASL gene cassettes containing either split UnaG (Fig. 4a) or Cre recombinase protein (Fig. 4b) with the requisite aaRS/tRNA pair into HEK293T and a reporter fibroblast cell line and used light to spatiotemporally control split-protein assembly. We also demonstrated LASL could be used to specify protein subcellular localization (Fig. 4c). Membrane-anchored ST or pSC was covalently ligated with complementary cytosolic pSC or ST protein upon light exposure.

### Additional applications

In addition to fluorescent and enzymatic split-protein complementation, we envision LASL can be extended to any class of protein for which alternative dimerizing chemistries have already proved effective, including for ligases, toxins and those involved in genome editing. Although we used ST/SC as our dimerizing domains, we anticipate that alternative isopeptide-forming chemistries, particularly those that are also dependent on a critical lysine residue, could be adapted for photomediated split-protein reconstitution. For example, SpyCatcher003 (ref. 35) or DogCatcher^36^ are two evolved variants of SpyLigation that have enhanced transamination kinetics and could be photocaged in a similar manner to LASL. A smaller ligation scar between the split-protein pairs can be achieved through strategies using a three-component system (e.g., SpyStapler^37^, SpyLigase^38^, SnoopLigase^39^, transglutaminase factor XIII (ref. 40), sortase^24^).

### Advantages over alternative approaches

LASL is a powerful technique that utilizes light to rapidly and irreversibly couple split-protein pairs in a highly specific, spatiotemporally controlled manner. Furthermore, the genetic encodability of the fusion pairs enables its application in living systems. Unlike chemically induced dimerization techniques that modulate the interaction between target proteins through small-molecule binding, LASL activation can be performed with precise 4D control; although small-molecule inducers can be temporally controlled in a dosage-dependent manner^7^, spatial regulation is difficult^41^ and compounds can be toxic^42,43^. LASL-mediated protein reconstitution overcomes these limitations as activation can be controlled in a highly specific manner in living cells and with virtually no background reaction.

LASL outperforms or overcomes many of the limitations presented by current optogenetic approaches for triggered protein assembly: (1) Current optogenetic strategies most typically utilize light-responsive protein pairs whose noncovalent photoassociation quickly reverses in the dark, requiring continuous illumination for sustained protein activation. LASL’s covalent protein coupling can be performed with short exposure times (typically less than 20 min at 20 mW cm^−2^ for complete reaction) and in an irreversible manner. (2) Most optogenetic proteins are large (>500 amino acids per dimerizing pair) whereas LASL leaves a much smaller molecular scar (~130 residues in total)^13^. (3) Previously developed tools respond to visible light (*λ* = 450–650 nm), which makes experiments difficult to carry out under standard laboratory conditions and further limits their application with common fluorophores in the visible light range (green/orange/red). LASL’s responsiveness to near-UV light enables it to be controlled in typical experimental settings with minimal precaution and alongside common fluorophores. (4) The high light responsiveness of prior strategies largely preclude their dose-dependent activation, whereas the extent of LASL can be precisely specified following well-behaved photokinetics. (5) While other optogenetic systems cannot be readily controlled in full 3D space, LASL’s multiphoton responsiveness affords a unique route toward full spatiotemporally controlled activation. (6) Though other tools have been reported for phototriggered protein ligation (e.g., BLISS^44^, split inteins^45^)—but notably not for split-protein reconstitution—these methods suffer from substantial dark reaction and background leakiness that is not observed with LASL. Collectively, LASL provides a route to precise and irreversible protein activation in full 4D that has been inaccessible by alternative strategies.

### Limitations and considerations

Although LASL provides many unique opportunities in photoactivating protein function via optical ligation of split pairs, it is certainly not without limitations. LASL’s dependence on genetic code expansion, which utilizes an ncAA that is not yet commercially available, and cotransformation/transfection of the aaRS/tRNA for protein expression adds complexity and places some limitations on its utilization in vivo. Additionally, as with any light-based chemistry, LASL’s reaction specification is limited to optically accessible sample regions; even with two-photon activation (which also requires a small-molecule photosensitizer in the current iteration), controlling activation within samples greater than 1 cm will probably prove challenging. Since near-UV light is employed for single-photon-based activation, light dosage should be carefully controlled to avoid unwanted damage through generation of reactive oxygen species^46,47^ or DNA oxidation^48^.

Although irreversible activation via LASL-mediated split-protein assembly is viewed as one of the technique’s biggest and most unique strengths, this irreversibility largely precludes its usage in applications involving sequential activation/deactivation-type studies. This limitation could potentially be addressed through future incorporation of a photocleavable protein (e.g., PhoCl^49^), permitting both activation and deactivation to be optically controlled within the same system.

As with all other inducible split-protein dimerization techniques, LASL’s success depends on identifying appropriate split sites that allow for their conditional reassembly; fragments must not spontaneously associate into a functional form and the ligated fragments must be spatially oriented in a manner that yields an active species. Despite these potential concerns, the large number of proteins that have been split for conditional assembly through various noncovalent techniques provides a starting point for irreversible protein activation via LASL.

### Expertise

The equipment used in the synthesis steps is accessible to standard chemistry facilities and most laboratories. Commercial routes are also available for custom chemical synthesis (e.g., that for Lys(*o*NB) or polyglycine peptides) that could simplify the procedure steps below. Compound characterization does require access to an NMR spectroscope and a mass spectrometer, but these instruments/services are common and should be available at most academic institutions. For peptide synthesis, an automated solid-phase peptide synthesizer is recommended but the synthesis can be carried out manually in the absence of such specialty equipment^50^. To design the plasmids for both bacterial and mammalian expression, the protocol provides a thorough step-by-step guide that can be supplemented with additional resources found on vendor websites. Cloned plasmid sequencing should be carried out by a suitable service facility and is usually inexpensive. For expression of the proteins in *E. coli* and mammalian cells, standard molecular cell biology and general tissue culture equipment are used in the protocol and can be implemented by most nonspecialized laboratories. Mammalian experiments should take place in suitable bioclassified laboratories. Depending on the resolution of patterning, a range of light sources at different prices can be used, including cheap light-emitting diode lights (US$10–100), moderately expensive collimated light sources ($100–10,000) or costly specialty lasers and microscopes ($100k–1M). In general, the cost and infrastructural investment of the light equipment is proportional to the photopatterning resolution.

### Experimental design

#### Procedure 1: Lys(*o*NB) synthesis and characterization

**Fig. 5 |:**
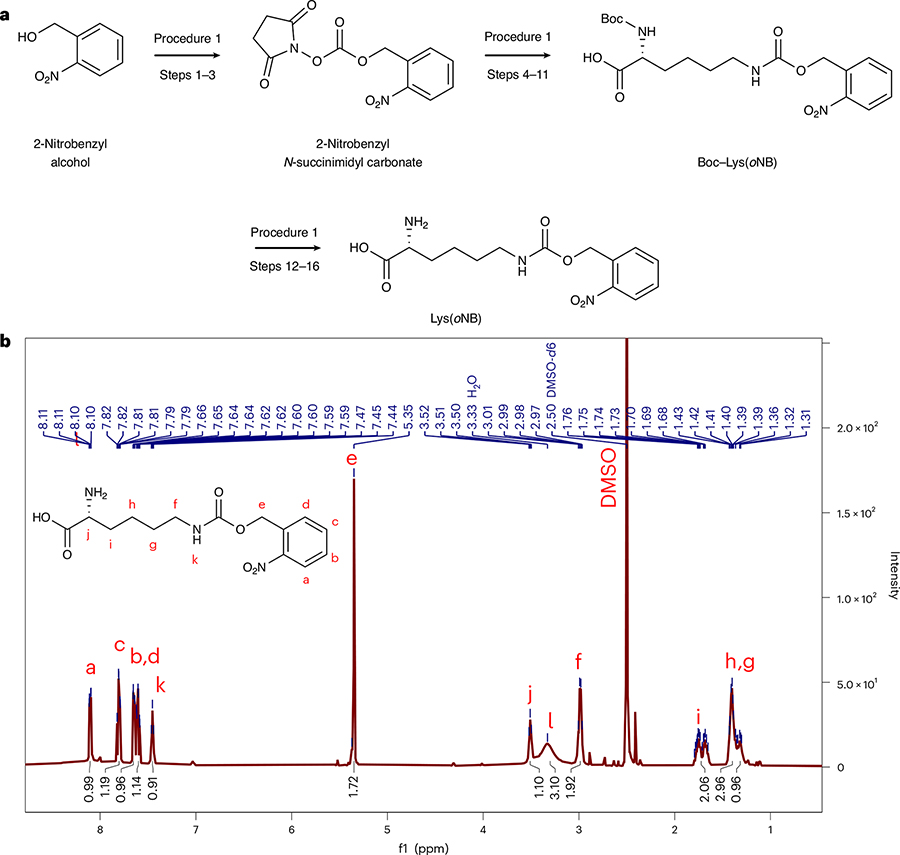
Synthetic route and characterization of Lys(*o*NB). **a**, Lys(*o*NB) is prepared through a three-step coupling reaction. **b,** ^1^H NMR spectra of Lys(*o*NB) in DMSO-*d*6. The spectral data and chemical shifts labelled a–k for Lys(*o*NB) can be found in the Anticipated Results section.

The implementation of LASL depends on the efficient incorporation of Lys(*o*NB) within pSC. This protocol describes a three-step reaction scheme for the synthesis of Lys(*o*NB) (Fig. 5). In the first step, 2-nitrobenzyl-*N*-succinimidyl carbonate is generated through a base-catalyzed reaction between *N*-*N*′-disuccinimidyl carbonate^51^ and 2-nitrobenzyl alcohol. The reaction can be completed in ~1 day, including both a 30 min reaction and a simple solvent extraction purification step of the intermediate. The second step is typically completed in 2 days and involves installing the nitrobenzyl group on a Boc-protected lysine (N_α_-Boc-L-lysine) and purification of the crude product through flash column chromatography. In the third step, an acid-mediated Boc deprotection reaction is carried out to yield the final Lys(*o*NB) product. The synthesis proceeds efficiently on the multi-gram scale with typical yields of product (~50%, 2 g) that can be used for many liters of protein expression (336 mg Lys(*o*NB) required per liter of expression). The chemicals used in the reaction are common and inexpensive (e.g., nitrobenzyl alcohol is $1.50/g), rendering the synthesis amenable to further scale-up; the complete reagent cost per reaction on the described scale is $10.

#### Procedure 2: design and cloning of LASL constructs

**Fig. 6 |:**
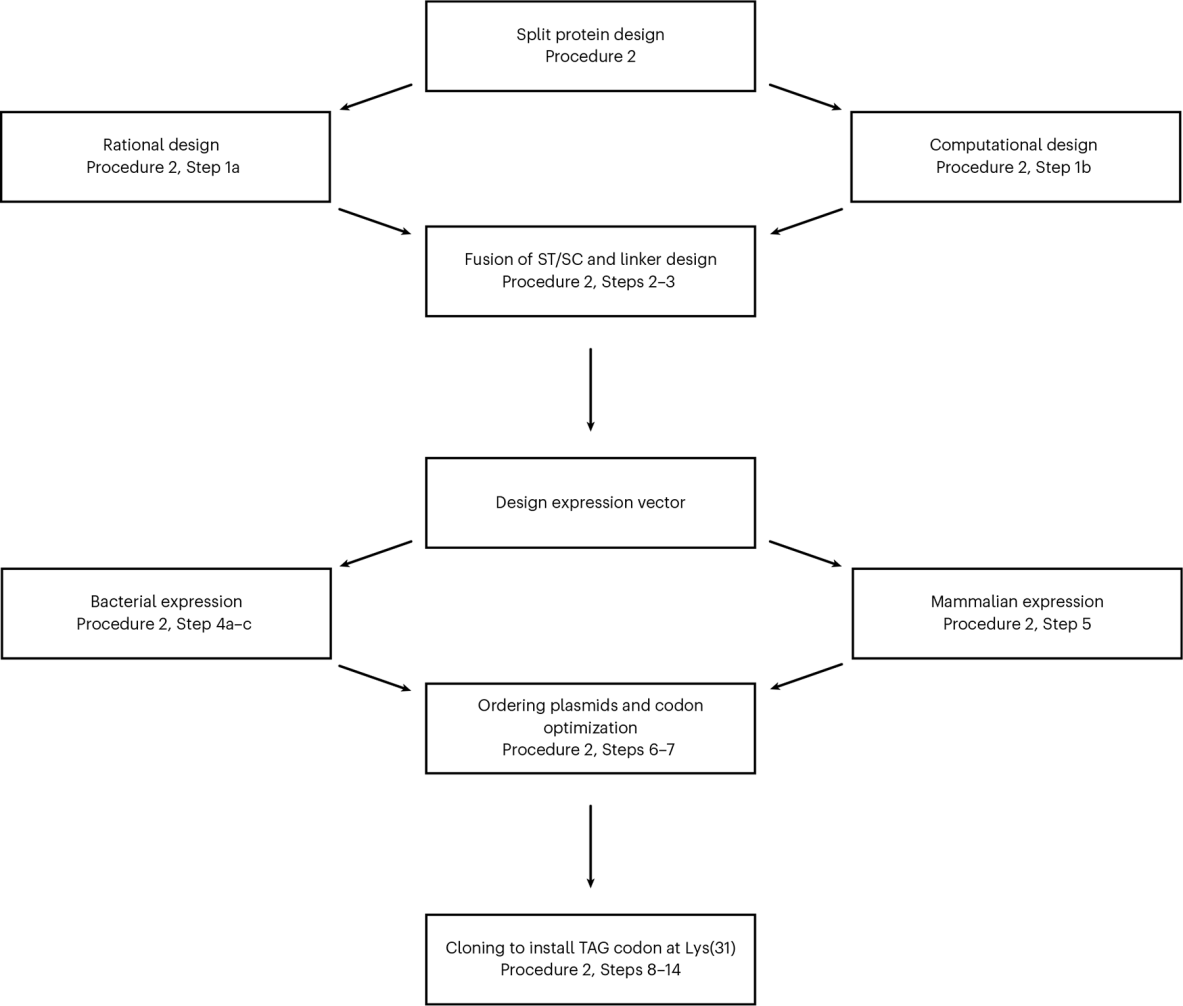
Workflow of LASL split-protein design. A split POI (generated either through rational or computational design) is genetically fused to ST/SC to create LASL constructs. LASL gene sequences can be inserted into either bacterial or mammalian-based expression plasmids. A simple cloning procedure can be used to install the TAG codon to generate the pSC variant.

Generating noncomplementing split proteins can be challenging, with the design process largely dependent on geometry, sterics and folding of the protein. Although there is no consensus on the optimal route to design split-protein systems, we provide a general workflow for doing so here (Fig. 6). Split sites can be determined through either rational or computational methods or a combination of both. We recommend first conducting a thorough literature search and using previously validated split sites as a starting point if they are available. If split sites have not been identified for a protein, computational tools such as AlphaFold^52^ and RoseTTAFold^53^ may aid in identifying split sites based on the protein structure. Furthermore, pipelines such as split-protein optimization by reconstitution (SPORT^54^) can be used to guide conditional split-protein design in a more automated fashion.

Once split sites for the POI have been generated, the N- or C-terminal fusion of ST and SC to each fragment (pSC–*x*POI and *x*POI–ST, where *x* is the N or C terminus) along with appropriate linkers that allow for conditional reconstitution without affecting the function of the target protein or dimerizing pair can be identified. We provide examples of linkers successfully used for LASL split-protein reconstitution and recommend these as a starting point. If generating alternative linkers, it is recommended to favor small and soluble amino acids (e.g., Ala, Gly, Ser) over bulky, hydrophobic amino acids (e.g., Pro, Ile, Phe, etc.)^55^. If the SpyLigation reaction is slow or inefficient, elongated linkers are recommended. Linker length can be further varied to tune the maximal activity of the LASL-reconstituted protein. We have also included examples of solubilizing factors that can be fused to ST fragments.

After the fusion pairs have been designed, the next step is to build the expression vector. We provide details on general plasmid vector design used for bacterial and mammalian expression of LASL constructs and considerations to take (e.g., antibiotic selection if cotransforming, codon optimization). Plasmids can be ordered through a reputable vendor or cloned into existing expression vectors in house. We recommend purchasing/cloning and validating functional protein assembly via SpyLigation using the WT SC pair; a simple site-directed mutagenesis procedure for inserting the amber TAG stop codon can be used to obtain the pSC required for LASL-based reconstitution (Procedure 2, Steps 8–14). Forward and reverse primers sequences for this point mutation are included.

#### Procedure 3: *E. coli* expression and characterizing activity

**Fig. 7 |:**
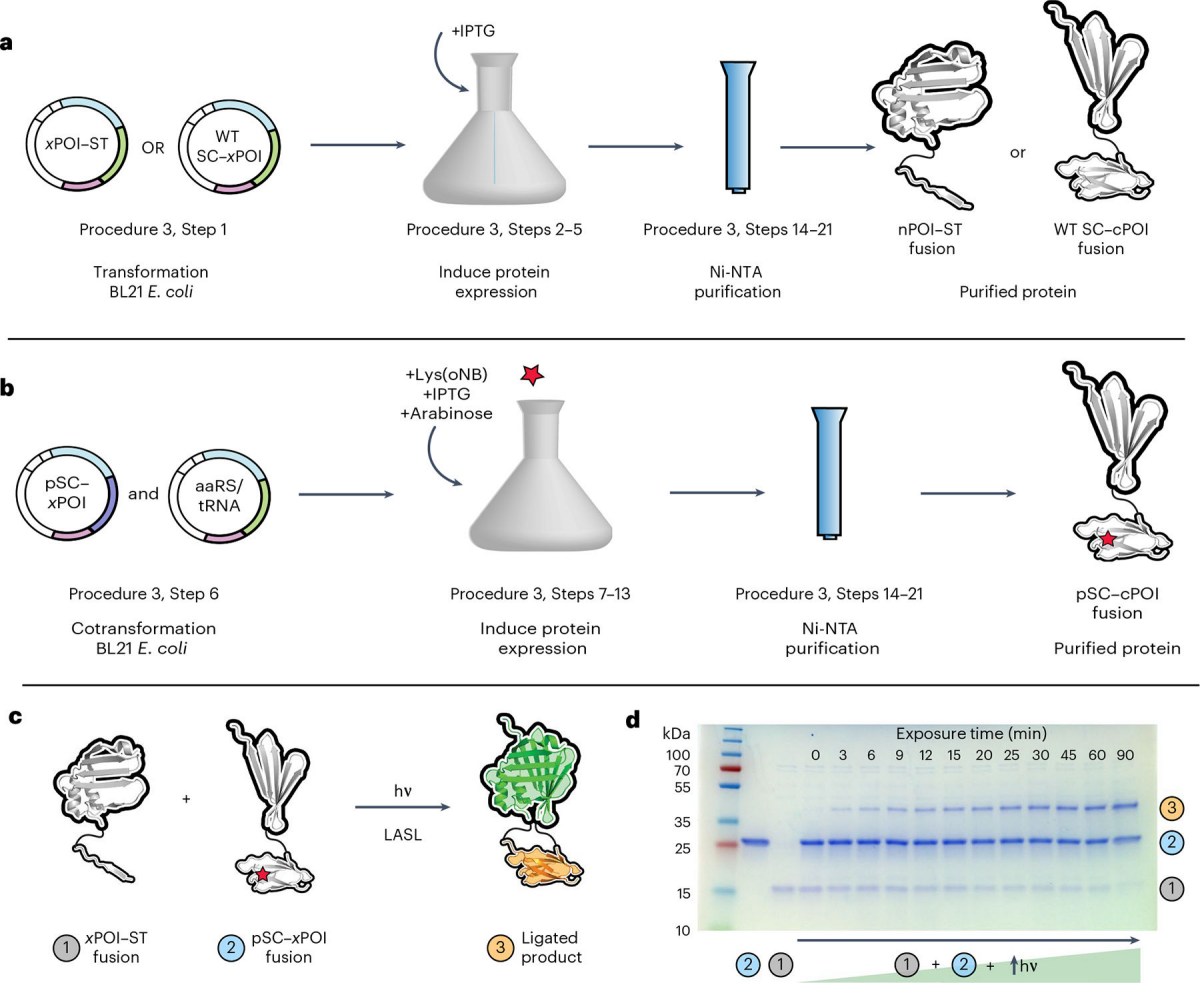
Schematic overview of the workflow for LASL *E. coli* protein expression. **a**, The WT SC and ST plasmids are transformed into Bl21(DE3) *E. coli* for recombinant protein expression. Expression of the SC or ST protein is accomplished by induction with IPTG. Proteins are purified through Ni-NTA affinity chromatography. **b**, The pSC and aaRS/tRNA plasmids are co-transformed into Bl21(DE3) *E. coli* and protein expression is induced by the addition of IPTG, arabinose and Lys(*o*NB) is added into the media for incorporation at pSC’s amber stop codon. The pSC protein is purified through Ni-NTA affinity chromatography. **c**, Covalent linkage of pSC–*x*POI (1, gray) and *x*POI–ST (2, blue) proteins is inhibited by the presence of Lys(*o*NB) at the critical lysine residue. pSC photoactivation allows for formation of the covalently ligated product (3, orange). **d**, The extent of LASL between pSC and ST can be characterized through an SDS–PAGE gel shift assay, where pSC/ST solutions are exposed to varying doses of light. Photokinetic rates can be assessed by quantifying band intensity of each species as labeled in **c**. **c** and **d** adapted from ref. 11, Springer Nature Ltd.

The genetically engineered bacterial ST and WT SC plasmids can be transformed into the *E. coli* Bl21(DE3) expression host and we provide a general protocol for protein production (Fig. 7a). Expression of the pSC mutant requires cotransformation of both the pSC vector and pEVOL–NBK-1 plasmid as well as the addition of the Lys(*o*NB) to the expression media (Fig. 7b). Given that amber suppression efficiency depends on the sequence around the amber codon^56,57^, which should be the same for all pSC constructs, other split-protein design aspects are more likely responsible in cases of low protein yields. Optimizing expression conditions (e.g., growth medium, temperature, *E. coli* strain, antibiotic concentration, incubation period) may help further increase protein production.

Purification of the LASL components expressed in *E. coli* is accomplished with a 6×-polyhistidine (6×His) tag, but essentially any purification tag can be theoretically utilized. Optimally, the purification tag should be placed on the C terminus of the pSC fragment such that only proteins for which the amber codon has been successfully suppressed (i.e., Lys(*o*NB) has been incorporated) will be isolated. In cases where C-terminal modification affects catalytic activity or results in poor solubility of the protein, incorporating the affinity tag on the N terminus will necessitate additional purification (e.g., size-exclusion chromatography) to separate the truncated and full-size pSC-containing fusion.

Purified LASL proteins can be characterized using a variety of techniques. SDS–PAGE is useful for elucidating the solubility of the proteins, sample purity of the elution fractions and amber suppression efficiency, which can be determined by image-based band intensity quantification of the truncated and full-length protein. Whole-protein LC–MS can also be used to determine protein identity and exposing a solution of the pSC fusion protein to near-UV light for 20 min at 20 mW cm^−2^ should yield a 179 dalton mass shift corresponding to successful photochemical cleavage of the *o*NB cage.

To test LASL-mediated reconstitution in solution, three critical components should be assessed: (1) proper ligation of the ST/SC, (2) restored function of the split protein upon reconstitution and (3) light-dependent split-protein reconstitution via LASL. To assess whether split-protein fragments properly participate in SpyLigation, equal molar amounts of the ST and SC fragments at various concentrations can be incubated together for nonkinetically limited times (several hours). Ligation extent can be determined with SDS–PAGE by quantifying the intensities of the disappearing bands from WT SC and ST along with the appearing bands of the corresponding SpyLigated product. An SDS–PAGE gel shift assay is also a useful tool for determining the absence or presence of ‘dark reaction’ for unexposed pSC samples. The first-order kinetic constant for pSC photoactivation can also be determined by exposing pSC to various amounts of light and quantifying bands in the SDS–PAGE gel (Fig. 7c,d). Assays specific to the split POI should be performed to verify successful restoration of protein function following SpyLigation; activity kits are commercially available for many common enzymes, though specific assays may need to be developed that are matched to the unique features of the activated protein.

#### Procedure 4: biomaterial incorporation and patterning

There is an expansive toolbox of techniques available to site-specifically modify proteins for biomaterial incorporation^58^. In this protocol, we highlight chemoenzymatically modifying pSC via sortase-based transpeptidation with a H-GGGGDDK(N_3_)-NH_2_ polyglycine probe, generating an azide-tagged species that can be incorporated into hydrogels formed via SPAAC reaction (e.g., between four-arm PEG tetrabicylononyne (PEG-tetraBCN) and a linear PEG-diazide). The single-step syntheses of the PEG-tetraBCN and PEG-diazide macromers utilizing commercially available starting materials can be carried out in parallel and are described in Boxes 2 and 3, detailing the solid-phase peptide synthesis and functionalization of the H-GGGGDDK(N_3_)-NH_2_ polyglycine probe. Since the functionalization of the macromers and polyglycine probe involve simple coupling reactions, various macromers and functionalization handles can be substituted in the reaction steps.

For sortase-mediated functionalization of pSC, three components are required: (1) pSC modified with the LPETG motif, (2) purified sortase A protein and (3) the polyglycine probe. During the genetic engineering process of the pSC plasmid for *E. coli* expression (Procedure 2), the LPETG sortase recognition sequence can be genetically incorporated at the C terminus before the polyhistidine purification tag. The complimentary ST protein does not require any additional modifications for in-gel reconstitution. The LPETG pSC and ST plasmids can be cloned, expressed, purified and characterized by following the same steps for *E. coli*-based protein expression described in Procedure 3. The sortase A expression plasmid is available from Addgene or the purified protein can be readily purchased from various vendors.

**Fig. 8 |:**
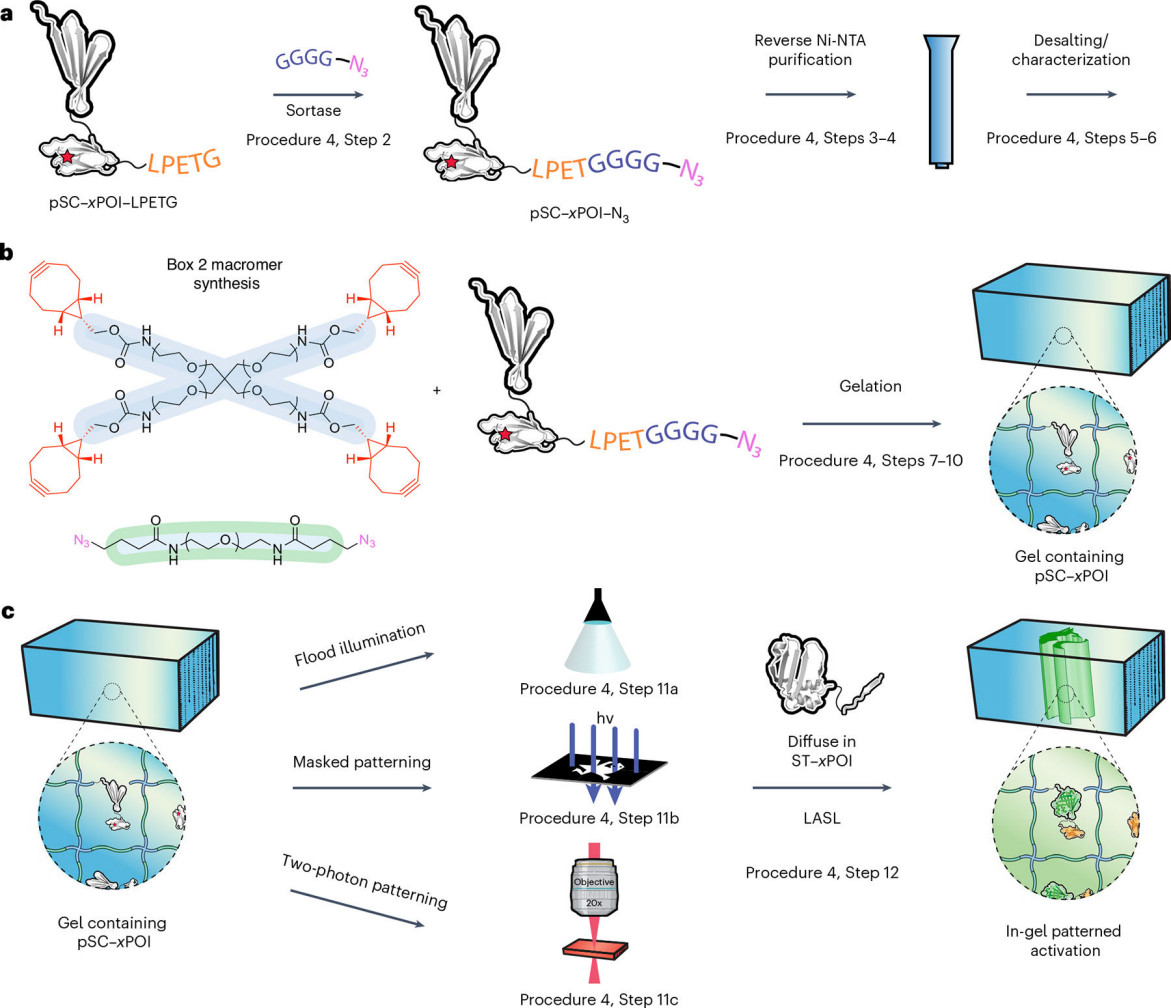
Schematic overview of the workflow for LASL biomaterial incorporation. **a**, Incorporation of a C-terminal sortase recognition motif on the pSC fusion (pSC–*x*POI–LPETG) enables biofunctionalization of the protein for biomaterial decoration through a sortase-mediated reaction. Sortagging is promoted by the addition of an azide-functionalized polyglycine probe (Box 3), which catalyses peptide ligation to the pSC and a simultaneous displacement of the 6×His-tag. The functionalized pSC–*x*POI–N_3_ is purified through reverse chromatographic isolation on Ni-NTA resin and desalted to remove excess peptide. **b**, Step-growth polymerization of PEG-tetraBCN and PEG-diazide (macromer synthesis is described in Box 2) in the presence of pSC–*x*POI–N_3_ form a SPAAC-based hydrogel uniformly decorated with the pSC. **c**, Patterning of the pSC-functionalized hydrogels can be accomplished by three photolithographic routes. Photoactivation permits spatiotemporally defined immobilization of complimentary ST constructs via LASL. **b** adapted from ref. 11, Springer Nature Ltd.

The sortase-based monotagging of the purified LPETG pSC protein with the azido–polyglycine peptide probe is carried out at 37 °C (Fig. 8a). Component conditions and ratios are described in Procedure 4, Step 2. During the reaction, sortase forms an acyl-enzyme intermediate with the sorting signal’s threonine residue and cleaves the C-terminal amino acids. The thioester is nucleophilically displaced by the H-GGGGDDK(N_3_)-NH_2_ polyglycine probe, which is present in excess. The modified pSC is purified through reverse Ni-NTA affinity chromatography; since the polyhistadine purification tag is removed during the sortagging reaction, the 6×His-tagged sortase and unmodified pSC protein remain bound to the Ni-NTA while the azide-functionalized pSC protein is isolated in the flow-through fraction. Sortagging efficiency can be determined through mass spectrometry and an SDS–PAGE gel upshift assay involving reaction with monofunctionalized methoxy–PEG (mPEG)–BCN.

To generate uniformly decorated pSC hydrogels formed through step-growth polymerization, the azide-functionalized pSC is first reacted with PEG-tetraBCN followed by the addition of the diazide cross-linker (Fig. 8b). The pSC hydrogels can be patterned using any of the photolithographic techniques described in Box 1 (Fig. 8c). Since SpyLigation should only occur at uncaged pSC sites, the ST-modified protein can be introduced within the hydrogel before or following photopatterning. Diffusive removal of any unreacted ST may further reduce background signal stemming from noncovalent association between the split-protein pairs. Genetic fusion of a fluorescent protein to the SpyTagged fragment can provide a visual readout for split-protein immobilization in the hydrogel. This strategy is beneficial when patterning growth factors or other proteins that lack intrinsic fluorescence, luminescence or colorimetric output.

#### Procedure 5: mammalian expression and characterizing activity

**Fig. 9 |:**
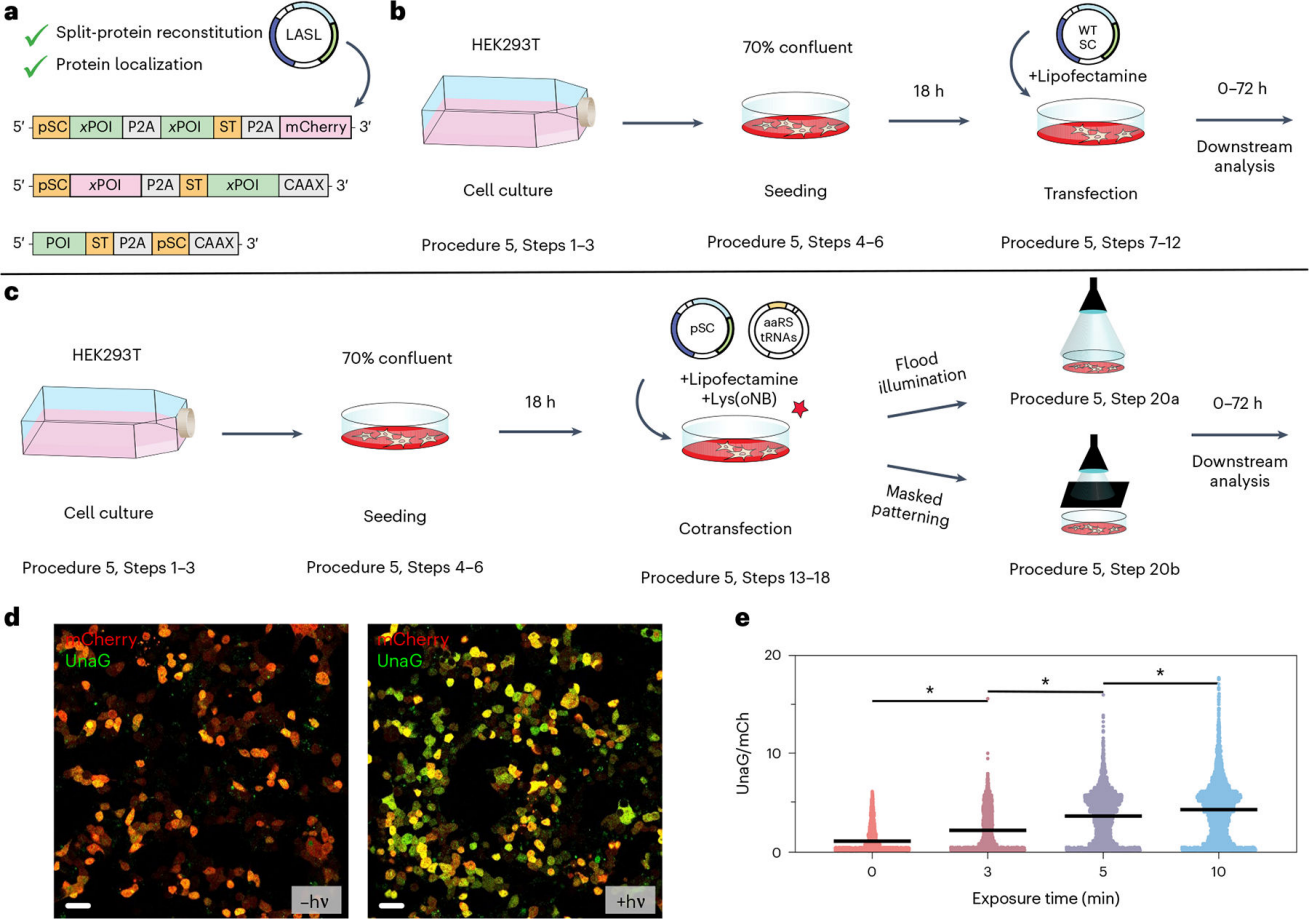
Schematic overview of the workflow for LASL mammalian protein expression. **a**, LASL polycistronic constructs for mammalian expression. **b**, Cultured HEK293T cells are seeded onto 35 mm glass-bottom dishes coated with gelatin. Cells are transfected with the WT SC and incubated until downstream analysis. **c**, For the LASL construct, seeded cells are cotransfected with the pSC and aaRS/tRNA gene casettes and incubated in growth media containing the Lys(*o*NB) for proper amber suppression. Intracellular LASL of transfected cells can be accomplished through two photolithographic routes before downstream analysis. **d**, Representative fluorescent images of transfected HEK293T cells (red) with (+hν) and without (−hν) light illustrate UnaG photoactivation (green) by LASL. **e**, Violin scatter plots of normalized UnaG/mCh ratios for individual cell UnaG/mCh signal treated with 0, 3, 5, 10 min of light (20 mW cm^−2^, *λ* = 365 nm) The asterisks denote conditions with statistically significant differences in signal (*P* < 0.0001, two-tailed unpaired *t*-tests). **d** and **e** adapted from ref. 11, Springer Nature Ltd.

In this section, we provide a procedure for delivering and expressing the WT SC or pSC fusion gene cassettes into HEK293T cells (Fig. 9). The protocol can be adapted for any desired mammalian cell line, though cell seeding densities, transfection conditions, incubation periods and light dosage conditions will need to be optimized. After designing the plasmids for mammalian expression (Procedure 2), transfection-grade DNA of the WT SC, pSC and pNeu–hMbPylRS–4xU6M15 plasmids should be generated using any standard DNA purification kit. Testing the WT SC cassette is important as it is helpful in determining whether the designed system can properly participate in SpyLigation and reconstitute protein function. As an auxiliary control, cells should also be transfected with a plasmid containing the intact POI.

Before transfection, cells should be seeded into 35 mm glass-bottom dishes and grown to 70% confluency. For exogenous delivery of the plasmids, we describe a transient transfection protocol using a Lipofectamine^™^ cationic-lipid reagent, but other reagents or approaches (e.g., stable transfection, nucleofection, viral transduction) can be utilized. Properly expressing the pSC variant requires cotransfection of the aaRS/tRNA plasmid and the addition of Lys(*o*NB) into the cell culture growth medium. An advantage of using transient transfection is that once the Lys(*o*NB) is removed from the growth medium, protein translation of the LASL components is terminated at the amber codon. Following transfection, cells should be incubated for a minimum of 24 h for sufficient protein expression. For the WT SC construct, downstream analysis can be performed following transfection. In the case of the pSC construct, light exposure experiments should be carried out in phosphate-buffered saline to reduce the formation of toxic photoproducts^59^. Photopatterning of the cells can be carried out at room temperature (20–22 °C) or under physiological conditions. After light exposure, cells can be incubated in Lys(*o*NB)-free medium, which will stop protein translation of the LASL construct at the amber stop codon.

The incubation period after light exposure before downstream analysis will vary. We observed stable protein signal of LASL expressed fragments for at least 72 h posttransfection. For LASL-based fluorescent protein localization experiments, we characterized cells 8 h after light exposure as the highest SC/ST ligation was observed at this timepoint. For UnaG split-protein reassembly, cells were characterized 3–6 h following light exposure. In the case of Cre recombinase activation, cells were incubated for 48 h before analysis. Including fluorescent tags on split-protein pairs may facilitate verification of proper protein expression and localization. Reporter-labeled cell lines with visually identifiable changes in phenotype (e.g., fluorescence, luminescence) in response to protein activity are also recommended.

## Materials

### Reagents

#### Procedure 1

- *N*,*N*′-Disuccinimidyl carbonate (Sigma, cat. no. 225827-5G)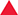 **CAUTION** *N,N*′-Disuccinimidyl carbonate is harmful upon inhalation or contact with skin and causes eye irritation.
- 2-Nitrobenzyl alcohol (ThermoFisher, cat. no. AAA1549614)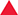 **CAUTION** 2-Nitrobenzyl alcohol is harmful upon inhalation or contact with skin and causes eye irritation.
- Triethylamine (ThermoFisher, cat. no. O4884-100)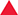 **CAUTION** Triethylamine is flammable, harmful upon inhalation or contact with skin and causes eye irritation.
- Acetonitrile (ThermoFisher, cat. no. A9964)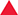 **CAUTION** Acetonitrile is acutely harmful upon inhalation or contact with skin and causes eye irritation.
- Ethyl acetate (ThermoFisher, cat. no. 6000575)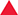 **CAUTION** Ethyl acetate is flammable, causes eye irritation and damage to organs.
- Anhydrous magnesium sulfate (MgSO_4_) (Sigma, cat. no. M7506-500G)
- Hexane (ThermoFisher, cat. no. H3024)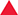 **CAUTION** Hexane is flammable, harmful upon inhalation or contact with skin and causes eye irritation.
- 1,4-Dioxane (ThermoFisher, cat. no. D111500)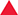 **CAUTION** Dioxane is flammable, harmful upon inhalation or contact with skin and causes serious eye irritation.
- Hydrochloric acid (HCl) (ThermoFisher, cat. no. S25358)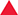 **CAUTION** HCl is corrosive and toxic.
- N_α_-Boc-L-lysine (ChemImpex, cat. no. MFCD00038203-25G)
- *N*,*N*-Dimethylformamide (DMF) (ThermoFisher, cat. no. MDX17276)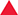 **CAUTION** DMF is acutely harmful upon inhalation or contact with skin and causes eye irritation.
- *N*,*N*-Diisopropylethylamine (DIEA) (ChemImpex, cat. no. 00141)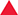 **CAUTION** DIEA is harmful upon inhalation or contact with skin and causes eye irritation.
- Diethyl ether (ThermoFisher, cat. no. AC123990050)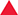 **CAUTION** Diethyl ether is flammable and harmful upon inhalation.
- Silica gel (Silicycle, cat. no. R10030B-25KG)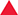 **CAUTION** Silica is harmful upon inhalation or contact with skin and causes eye irritation.
  Silica should be handled with an appropriate face mask for particulates.
- Silica thin layer chromatography (TLC) plates (Silicycle, cat. no. TLA-R10011B-323)

#### Procedure 2 (additional reagents)

- Phusion Master Mix (ThermoFisher, cat. no. F531S)
- DpnI (NEB, cat. no. R0176S)
- Carbenicillin (ThermoFisher, cat. no. BP26485)
- Kanamycin (ThermoFisher, cat. no. BP9065)
- Chloramphenicol (ThermoFisher, cat. no. AC227920250)
- NEB 10-beta competent *E. coli* (NEB, cat. no. C3019I)
- Yeast extract (ThermoFisher, cat. no. BP9727500)
- Tryptone (ThermoFisher, cat. no. BP1421-500)
- Agar (ThermoFisher, cat. no. BP9724-500)
- Sodium chloride (ThermoFisher, cat. no. S2711)

#### Procedure 3 (additional reagents)

- pEVOL–NBK-1 plasmid for the expression of *M. mazei* pyrrolysine aminoacyl–tRNA synthetase/tRNA pair (PylRS/tRNAPyl) in *E. coli* (Addgene, cat. no. 207639)
- SC, pSC and ST LASL plasmids for expression in *E. coli* for in solution studies (for design and ordering of plasmids, see Procedure 2)
- Isopropylthio-β-galactoside (IPTG) (Gold Biotechnology, cat. no. I2481C50)
- Arabinose (ThermoFisher, cat. no. A0515250G)
- BL21(DE3) Competent *E. coli* (ThermoFisher, cat. no FEREC0114)
- Glycerol (ThermoFisher, cat. no. BP9727500)
- Potassium dihydrogen phosphate (KH_2_PO_4_) (ThermoFisher, cat. no. AC212595000)
- Dipotassium phosphate (K_2_HPO_4_) (ThermoFisher, cat. no. P290500)
- Ni-NTA superflow resin (Gold Biotechnology, cat. no 4156)
- Tris Base (ThermoFisher, cat. no. BP1521)
- Imidazole (ThermoFisher, o3196500)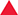 **CAUTION** Imidazole is acutely harmful upon inhalation or contact with skin and causes severe eye irritation.
- Complete ethylenediaminetetraacetic acid (EDTA)-free protease inhibitor cocktail tablets (Fisher, cat. no. PIA32965)
- SDS (ThermoFisher, cat. no. PI28312)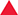 **CAUTION** SDS is acutely harmful upon inhalation or contact with skin and causes severe eye irritation.
- SDS–PAGE gel (BioRad, cat. no. 4561106)
- 2× Laemelli sample buffer (BioRad, cat. no. 1610737)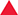 **CAUTION** Buffer is acutely harmful upon inhalation or contact with skin and causes severe eye irritation.
- Glycine (ThermoFisher, cat. no. BP381500)
- Precision Plus Protein Dual Color Standards (BioRad, cat. no 1610374)
- Formic acid (FA) (ThermoFisher, cat. no. A11750)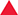 **CAUTION** FA is flammable, harmful upon inhalation or contact with skin and causes eye irritation.
- Sodium hydroxide (NaOH) (ThermoFisher, cat. no. BP359500)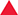 **CAUTION** NaOH is corrosive and toxic.

#### Procedure 4 (additional reagents)

- SC–LPETG, pSC–LPETG and ST LASL plasmids for expression in *E. coli* for biomaterial incorporation (for design and ordering of plasmids, see Procedure 2)
- pET30b-7M SrtA (SrtA_7M_) plasmid (Addgene, cat. no. 51141)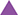 **CRITICAL** The SrtA_7M_ is a heptamutant of the original sortase A and does not require the addition of calcium for sortagging. As an alternative, the purified protein can be ordered from various vendors (e.g., BPS Bioscience, cat. no. 71048)
- Rhodamine B (ThermoFisher, cat. no. AAA1357230)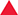 **CAUTION** Rhodamine B causes severe skin and eye irritation. Acute hazard to aquatic environments.
- Rain-X (Rain-X, cat. no. 800002243)

#### Procedure 5 (additional reagents)

- WT SC and pSC plasmids for expression in mammalian cells (for design and ordering of plasmids, see Procedure 2)
- pNeu–hMbPylRS–4xU6M15 plasmid for the expression of *M. mazei* pyrrolysine aminoacyl–tRNA synthetase/tRNA pair (PylRS/tRNAPyl) in mammalian cells (Addgene, cat. no. 105830)
- HEK293T cells (ATCC, CRL-1573; RRID: CVCL_0063) or other mammalian cell lines of interest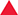 **CAUTION** Cell lines should be routinely checked for mycoplasma.
- Gelatin (ThermoFisher, cat. no. 5025935)
- Lipofectamine 3000 (ThermoFisher, cat. no. L3000008) or other transfection reagent of choice
- Dulbecco’s modified Eagle medium (DMEM) (+4.5 g/l-glucose, +l-glutamine, −sodium pyruvate) (ThermoFisher, cat. no. 11965118)
- Penicillin–streptomycin (ThermoFisher, cat. no. 15140122)
- Fetal bovine serum (FBS) (Sigma, cat. no. F0926-500ML)
- Hanks Buffered Salt Solution (HBSS) (Gibco, cat. no. 14025076)
- Trypsin–EDTA solution (ThermoFisher, cat. no. 25200072)
- EndoFree Plasmid DNA Maxi Kit (Omega Biotek, cat. no. NC0768143)
- Opti-MEM reduced serum medium (Gibco, cat. no. 31985062)

### Equipment

#### Procedure 1

- Fume hood (Supreme Air, Kewaunee Scientific Corporation)
- Weighing balance (Mettler Toledo, cat. no. 30029077)
- Spatulas (Fisher Scientific, cat. no. 14373)
- Glassware (round-bottomed flasks, Erlenmeyer flasks, beakers, dishes) (Fisher Scientific)
- Buchner funnel (Fisher Scientific)
- Separatory funnel (Fisher Scientific)
- Qualitative filter paper (Cytiva, cat. no. 1001045)
- Magnetic stir plate (Fisher Scientific, cat. no. SP88854100)
- Magnetic stir bars (Fisher Scientific, cat. no. 14-513-51
- Schlenk link (Thomas Scientific, cat. no. M411003)
- Nitrogen gas (Linde Gas & Equipment Inc., cat. no. NI K)
- Rotary evaporator (Büchi Rotovapor R-3 equipped with a V-700 vacuum pump, V-855 vacuum controller and a Welch 1400 DuoSeal Belt-Drive high vacuum pump)
- Lyophilizer (LABCONCO FreeZone 2.5 Plus with a LABCONCO rotary vane 117 vacuum pump)
- Mass spectrometer (ThermoFisher, Linear Trap Quadrupole Orbitrap Xcalibur 2.0 DS)
- NMR spectrometer (Bruker)
- NMR tubes (Fisher Scientific, 16800555)
- 50 mL conical tubes (ThermoFisher, cat. no. 12-565-271)
- 500 mL chromatography column 40 mm inner diameter (Fisher Scientific, cat. no. 31-500-913)

#### Procedure 2 (additional equipment)

- Temperature-controlled shaking incubator (ThermoFisher, Model MaxQ 4000)
- NanoDrop spectrophotometer (ThermoFisher Model 2000C)
- PCR tubes (Fisher Scientific, cat. no. 14230210)
- Thermocyler (Bioer LifeECO)
- Sterile syringes, filters, needles (Fisher Scientific)

#### Procedure 3 (additional equipment)

- Microcentrifuge tubes (BrandTech, cat. no. 780420)
- Shaker flasks (PYREX, cat. no. 4446500)
- Tabletop centrifuge (Thomas Scientific, cat. no. 75007213)
- Floor centrifuge (Beckman Coulter)
- pH meter (Fisher Scientific, cat. no. 13636XL150)
- Sonicator (ThermoFisher Model 505 Soni Dismembrator)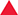 **CAUTION** Sonicator should be in a soundproof box or ear protection should be worn.
- SDS–PAGE system (BioRad)
- Scanner (Azure Model 600 AZI600)
- Vials for LC–MS (Waters, cat. no. 186000385C)
- LC–MS (AB Sciex 5600 QTOF)

#### Procedure 4 (additional equipment)

- Glass slides, 75 mm × 25 mm (Chemglass Life Sciences, cat. no. CGQ064001)
- Silicone rubber spacer (0.5 mm thick) (McMaster-Carr, cat. no 87315K61)
- 100 mm Petri dish (ThermoFisher, cat. no. FB0875712)
- Positive displacement pipette (Gilson, cat. no. FD10002)
- Zeba Spin Desalting Column (ThermoFisher, cat. no PI89882)
- Radiometer (series 9811–50; Cole-Parmer)
- UV light source (Lumen Dynamics OmniCure S1500 Spot UV Curing system with an internal 365 nm filter)
- Chrome photomask (PhotoSciences)
- Multiphoton microscope (ThorLabs Bergamo II)

#### Procedure 5 (additional equipment)

- Standard equipment for mammalian cell culture
- Biological safety cabinet (Thermo Scientific, cat. no. 1385)
- CO_2_ cell culture incubator set to 37 °C with 5% CO_2_ (ThermoFisher)
- Water bath set to 37 °C (ThermoFisher)
- Centrifuge (Fisher Scientific, cat. no. 01257134)
- T25 flasks (Fisher Scientific, cat. no. 12565351)
- Hemacytometer (Hausser Scientific, cat. no. 3520)
- 35 mm glass-bottom dishes, 14 mm glass microwell size (Cellvis, cat. no. NC0794151)

### Software

- Autodesk Netfabb Premium 2023.1 (https://www.autodesk.com)
- MATLAB R2019a (https://www.mathworks.com/products/matlab.html)

### Reagent setup

#### Procedure 1

- Saturated NaCl solution: dissolve 36 g of NaCl in 100 mL H_2_O
- Brine solution: mix 100 mL of H_2_O with 20 mL of saturated NaCl solution
- 4 M HCl in 1,4-dioxane: slowly add 10 mL 12 M HCl to 20 mL 1,4-dioxane in a fume hood

#### Procedure 2

- Carbenicillin (100 mg mL^−1^): dissolve 1 g carbenicillin in 10 mL H_2_O and filter sterilize. Store at −20 °C
- Kanamycin (30 mg mL^−1^): dissolve 0.3 g of kanamycin in 10 mL H_2_O and filter sterilize. Store at −20 °C
- Chloramphenicol (30 mg mL^−1^): dissolve 0.3 g of chloramphenicol in 10 mL 70% ethanol. Store at −20 °C
- Miller’s lysogeny broth (LB) medium (10 g L^−1^ tryptone, 5 g L^−1^ yeast extract, 10 g L^−1^ NaCl): Add 10 g tryptone, 5 g yeast extract and 10 g sodium chloride to 1 L H_2_O and sterilize by autoclaving. If required, antibiotics can be added to a final concentration of 30 μg mL^−1^ for kanamycin, 30 μg mL^−1^ for chloramphenicol or 100 μg mL^−1^ for carbenicillin
- LB agar plates (10 g L^−1^ tryptone, 5 g L^−1^ yeast extract, 10 g L^−1^ NaCl, 15 g L^−1^ agar): add 10 g tryptone, 5 g yeast extract, 10 g NaCl and 15 g agar to 1 L H_2_O before autoclaving. To prepare plates, allow the medium to cool to 50 °C, then add desired antibiotic stock, mix by gentle swirling and pour 30 mL into each sterile Petri dish (100 mm diameter). The final antibiotic concentrations should be 100 μg mL^−1^ carbenicillin, 30 μg mL^−1^ chloramphenicol and 30 μg mL^−1^ kanamycin. For double selection plates, add both relevant antibiotics

#### Procedure 3

- IPTG (1 M): dissolve 0.238 g of IPTG in 10 mL H_2_O and filter sterilize through a 0.22 μm syringe filter. Store 1 mL aliquots for up to 1 year at −20 °C
- Arabinose (10% (wt/wt)): dissolve 1 g arabinose in 10 mL H_2_O and filter sterilize through a 0.22 μm syringe filter. Store at room temperature
- 0.5 M NaOH: dissolve 40 mg NaOH solid in 1 mL H_2_O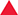 **CAUITION** NaOH is corrosive and toxic.
- 1 M HCl: mix 0.08 mL 12 M HCl with 0.92 mL H_2_O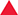 **CAUTION** HCl is corrosive and toxic.
- Phosphate buffer (10×): add 1.5 g KH_2_PO_4_ and 8.15 g K_2_HPO_4_ to 65 mL of H_2_O and sterilize by autoclaving. Allow the solution to cool to room temperature before adding to Terrific Broth medium
- Terrific Broth medium (24 g L^−1^ yeast extract, 20 g L^−1^ tryptone, 4 mL L^−1^ glycerol, 17 mM KH_2_PO_4_, 72 mM K_2_HPO_4_): for a 125 mL expression volume, mix 3 g of yeast extract, 2.5 g of tryptone, 1 mL of glycerol and 112.5 mL of H_2_O in a 500 mL shaker flask and sterilize by autoclaving. Allow the solution to cool to ~60 °C and add 12.5 mL of sterile 10× phosphate buffer
- Lysis buffer: prepare a solution of 20 mM Tris, 50 mM NaCl and 10 mM imidazole, pH 7.5. Store at room temperature
- Wash buffer: prepare a solution of 20 mM Tris, 50 mM NaCl and 20 mM imidazole, pH 7.5. Store at room temperature
- Elution buffer: prepare a solution of 20 mM Tris, 50 mM NaCl and 250 mM imidazole, pH 7.5. Store at room temperature
- SDS–PAGE running buffer: to make a 10× stock, combine 30.3 g Tris, 144.4 g glycine and 10 g SDS. Add distilled H_2_O until the volume is 1 L. Mix 100 mL of 10× stock and 900 mL H_2_O to make the final 1× SDS–PAGE running buffer
- 0.1% FA in water: add 1 mL FA to 999 mL of water
- 0.1% FA in acetonitrile: add 1 mL FA to 999 mL of acetonitrile

#### Procedure 4

- 50 μM Rhodamine B: dissolve 23.951 mg of Rhodamine B in 500 mL PBS. Store solution at room temperature covered from light
- 2P patterning chamber: cut a 10 mm circle inside of a 25 mm × 25 mm rubber spacer. Apply clear nail polish to one side of the rubber and attach to a glass slide

#### Procedure 5

- 1% Gelatin solution: dissolve 1 g of gelatin in 100 mL H_2_O and filter sterilize. Store at 4 °C
- HEK293T growth media: supplement DMEM medium with 10% (vol/vol) FBS and 1% (vol/vol) penicillin–streptomycin. Complete DMEM medium can be stored at 4 °C for several months
- 2.5 mM Lys(*o*NB) growth media: working solutions of media containing the ncAA are prepared freshly before the addition to cells. Prepare a 2.5 mM solution by dissolving the Lys(*o*NB) in 0.5 M NaOH and add to complete growth media. Add an equivalent amount of 1 M HCl. Media containing ncAA should not be stored for more than a few days as ncAA can precipitate
- LASL plasmids: generate the desired WT and pSC plasmids for mammalian expression as described in Procedure 2
- Transfection-grade DNA of expression vectors: isolate transfection-grade DNA of the WT SC, pSC and pNeu–hMbPylRS–4xU6M15 plasmids using a Maxi purification kit or other plasmid isolation kit according to the manufacturer’s protocol. Elute DNA in cell culture-grade water and dilute to 1 μg μL^−1^. Store at −20 °C.

## Procedure 1: Lys(*o*NB) synthesis

● TIMING <5 days

### Synthesis of 2-nitrobenzyl-*N*-succinimidyl carbonate

1. Mix 4.64 g disuccinimidyl carbonate (18.1 mmol, 1.1 equiv.), (16.3 mmol, 1 equiv.), 3.4 mL of triethylamine (24.5 mmol, 1.5 equiv.) and 30 mL acetonitrile in a dry 500 mL round-bottom flask. Dissolve 2.5 g of 2-nitrobenzyl alcohol in 20 mL acetonitrile and add dropwise to the mixture. Purge the flask with nitrogen and let the reaction run at room temperature for 30 min.
  ◆ TROUBLESHOOTING
2. To extract the reaction from Step 1, add 100 mL of ethyl acetate then transfer the organic mixture into a 500 mL separatory funnel. Wash the combined organic layer with 100 mL of 3× brine solution. The ethyl acetate organic phase with the product will be the lighter, upper layer.
3. Collect the organic fraction in a 250 mL round-bottom flask and concentrate the crude material under reduced pressure with a rotary evaporator to obtain an off-white solid product (2-nitrobenzyl-*N*-succinimidyl carbonate, ~4.11 g, 85% yield).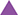 **CRITICAL STEP** Removing excess solvent may require a high vacuum with a liquid nitrogen cold trap. Check the product by TLC plate by dissolving a small amount of product in a 1:1 ethyl acetate/Hex mixture. To remove contaminants, the product may be washed by adding 1–3 mL of ethyl acetate to the flask, swirl for 1–2 min, pipette off the ethyl acetate and concentrate the material under reduced pressure with rotary evaporator (silica, 1:1 ethyl acetate/Hex, *R*_f_ ~0.4).

### Synthesis of Lys(*o*NB)

- Dissolve 4.11 g of the purified 2-nitrobenzyl-*N*-succinimidyl carbonate (14 mmol, 1 equiv.) in 30 mL of DMF in a round-bottom flask. Add 3.78 g N_α_-Boc-L-lysine (15.4 mmol, 1.1 equiv.) and 10.73 mL DIEA (61.4 mmol, 4 equiv.) to the flask and stir overnight at room temperature.
- Distill the reaction mixture under reduced pressure using with the rotary evaporator using a high vacuum pump to remove DMF.
- To extract the reaction from Step 5, add 50 mL of ethyl acetate then pour the organic mixture into a 500 mL separatory funnel. Add 100 mL brine solution.
- Collect the organic fraction in a 500 mL round-bottom flask. Repeat the extraction with 50 mL of EtOAc 2× and collect the organic layer. Concentrate the crude material under reduced pressure with a rotary evaporator to obtain a light brown oil. Check the product by TLC plate (silica, 1:1 Hex/(ethyl acetate + 1% acetic acid), *R*_f_ ~0.05).
- To purify the crude product, load 6 inches of silica slurry in 1:1 Hex/(ethyl acetate + 1% acetic acid) onto a 500 mL column. Add 2 cm of sand to the top.
- Dissolve the crude material from Step 7 in 1:1 Hex/(ethyl acetate + 1% acetic acid) and load as a liquid onto the column in Step 8.
  ◆ TROUBLESHOOTING
- Run a 1:1 Hex/(ethyl acetate + 1% acetic acid) solution through the column (~1.5 CV) and switch to 100% (ethyl acetate + 1% acetic acid) once the product begins eluting. Check each collected fraction by TLC plates (silica, 1:1 Hex/(ethyl acetate + 1% acetic acid), *R*_f_ ~0.05).
- Concentrate the elution fractions containing the desired product in a round-bottom flask using the rotary evaporator to obtain a light brown oil (Boc-protected Lys(*o*NB), ~4.09 g, 69% yield).
- To deprotect the product, add 3 mL 1,4-dioxane and 30 mL of 4 N HCl in 1,4-dioxane dropwise to the Boc-protected Lys(*o*NB). Stir the reaction at room temperature for 2.5 h.
- Concentrate the reaction under reduced pressure using the rotary evaporator to obtain a light brown oil.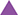 **CRITICAL STEP** Removing excess solvent may require a high vacuum with a liquid nitrogen cold trap.
- Precipitate the concentrated product from Step 13 by adding 15 mL of cold diethyl ether to the flask three times. The off-white solid Lys(*o*NB) should precipitate out within a few minutes. Transfer the solid to a 50 mL conical tube, and spin down at 4,000*g* for 5 min. Pipette off excess diethyl ether and dry the solid by blowing nitrogen over the product.
- Lyophilize the product overnight to remove remaining solvent and obtain the final Lys(*o*NB) product (~2.08 g, 51.4% yield).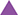 **CRITICAL STEP** The Lys(*o*NB) should be stored at −20 °C and protected from light. Under these conditions, the product should be stable for at least a year.
- Characterize the product using ^1^H NMR and mass spectrometry. For NMR, weigh 5 mg of Lys(*o*NB) and dissolve in 500 μL of DMSO d6.
  - Transfer the solution into an NMR tube and obtain NMR data of the compound. Use chemical shifts and integration of peaks to confirm the chemical structure
  - For mass spectrometry, weigh out 1 mg of Lys(*o*NB), dissolve in 500 μL of acetonitrile and transfer the solution to a mass spectrometry vial. Analyze the product using linear trap quadrupole (LTQ)–MS in positive mode

## Procedure 2: split-protein design

● TIMING 1–4 days

### Designing split proteins

1. Identify a desired POI and split site to generate the N- and C-terminal fragments (nPOI and cPOI, respectively) by either:
  - Rationally determining the split site using previously validated split-protein pairs in literature
  - Computationally generating nonself-reconstituting split-protein fragments by using sequence (http://www.uniprot.org/) and crystal structures (https://www.rcsb.org). RosettaRemodel (https://www.rosettacommons.org/docs/latest/application_documentation/design/rosettaremodel) and AlphaFold (https://alphafold.com) can be used to predict structures of the designed split domains
2. Using the nPOI and cPOI fragments generated, determine the arrangement of ST and SC at the N- or C-terminal using ST/SC crystal structure (https://www.rcsb.org/4MLI) and SpyInfo/SpyBank database. The amino acid sequence for ST and SC are listed in Table 1 with SC’s critical Lys(31) bolded.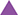 **CRITICAL STEP** There are four possible configurations for each fusion pair (ST–nPOI, nPOI–ST, ST–cPOI, cPOI–ST and SC–nPOI, nPOI–SC, SC–cPOI, cPOI–SC). We recommend starting with the experimentally and computationally validated arrangement nPOI–ST and pSC–cPOI.
3. Insert a linker between ST/SC and each split-protein fragment with the following in mind:
  - We recommend using the GGSGGGGSGGS linker between ST and GGSGGGSGG for SC protein fusions. Glycine/serine linkers typically have balanced flexibility, solubility and protease resistance
  - If generating alternative linkers, avoid using Asp or Asn residues near the C-terminal side of SC, which could promote self-reaction. Linker examples can also be found in the SpyBank database

### Designing plasmids for bacterial expression

- Once the orientation of the split-protein components has been determined, plasmid expression vectors can be designed for the SC, pSC and ST. Examples are provided in Table 2.
  - ST fragment *E. coli* expression vector: any suitable expression vector can be used. We recommend a pET-29a vector with kanamyacin resistance, P15A origin of replication, IPTG-inducible promoter and hexahistidine tag for purification
  - WT and photocaged SC fragment *E. coli* expression vector: the expression vector containing the SC fusion construct should be compatible with the pEVOL–NBK-1 vector, which encodes chloramphenicol resistance, contains the P15A origin of replication and araBad/glynS’ promoters. In general, we recommend a pET-21a vector with carbenicillin resistance, P15A replication, IPTG promoter and hexahistidine tag for purification
  - Optional sequence for hydrogel incorporation: a carboxy sortase recognition motif, LPETG, can be included at the C terminus of the SC gene insert before the 6×His-tag for downstream chemoenzymatic modification. The nucleotide sequence for LPETG is CTTCCAGAAACCGGC

### Designing plasmids for mammalian expression

- For mammalian expression ST/SC/POI fragments should be cloned into a single mammalian expression vector, with each fusion pair separated by a P2A sequence, with an optional fluorescent control added to the end. Table 3 summarizes a list of previously validated plasmid expression systems. The pSC expression vector should be compatible with the aaRS/tRNA plasmid (pNeu–hMbPylRS–4xU6M15).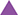 **CRITICAL STEP** Optimally, the pSC fusion pair should be expressed first; incomplete amber suppression will lead to unequal expression of the fragments. For transfection of HEK293T cells, a pcDNA3.1 vector with a cytomegalovirus (CMV) promoter was used. Promoter selection will vary by cell type. If stable transfection is desired, the resistance gene on the pSC expression vector should be orthogonal to the resistance of the aaRS/tRNA plasmid. Additionally, although P2A cleavage has been shown to be highly efficient, cleavage between fusion pairs may vary depending on the construct, and noncleaved proteins may contribute to background association.

### Ordering plasmids

- Each amino acid sequence should be codon optimized with the following considerations:
  - Codon optimize sequences for the species intended for expression; however, the original ST/SC codons without further optimization are recommended and listed in Table 4. If ST is located on the N terminus, two optimized sequences for vectors containing T5 or T7 promoters are listed below. SC’s critical Lys(31) is bolded
  - No amber codons should be included in the plasmid except the pSC amber stop codon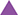 **CRITICAL STEP** Verify the terminal stop codon is not the same sequence as the amber stop codon.
- Order fully cloned plasmids from a reputable vendor. Free technical support, including codon optimization tools for expression hosts, are available through GenScript and Twist Bioscience, among others.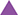 **CRITICAL STEP** Alternatively, Gibson assembly of the desired linearized vector plasmid in conjunction with the DNA gene fragment can be performed to generate LASL constructs for a more cost-effective method. Although both the WT SC and pSC plasmids may be ordered, the amber stop codon can be installed on the WT SC’s Lys(31) using Procedure 2, Steps 8–14, which may be a more cost-effective option. It is highly recommended to have the WT plasmid as a control to verify the designed fusion protein properly expresses.

### Installing TAG at pSC’s Lys(31)

- To generate the pSC plasmid for Lys(*o*NB) incorporation, install the amber stop codon (TAG) at SC’s Lys(31) through site-directed mutagenesis using the PCR amplification protocol below.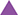 **CRITICAL STEP** The procedure and primers listed below can be used for generating both the bacterial and mammalian pSC plasmid as the SC nucleotide sequence is the same irrespective of expression host.
- Order the sequence for overlapping forward and reverse primers each containing the amber stop codon TAG in place of Lys(31) (SpyCatcher-SDM-Forward and SpyCatcher-SDM-Reverse) listed in Table 5 (Integrated DNA Technologies). Dilute the manufactured primers to a final concentration of 100 μM in water. Store at −20 °C. Primers are stable for at least a year under these conditions.
- Prepare a 50 μL PCR amplification reaction for each construct and aliquot the reaction mixture into PCR tubes as follows:

**Table:** 

Component	Volume	Final concentration
Phusion master mix (2×)	25 μL	1×
5’ Primer (10 μM stock)	1 μL	1 μM
3’ Primer (10 μM stock)	1 μL	1 μM
Template SC DNA (25 ng μL^−1^)	1 μL	25 ng
Water	22 μL	—
- Run the reaction mixture in a thermocycler using the following conditions:

**Table:** 

Cycle number	Denature	Anneal	Extend
1	95 °C, 2 min		
2–26	95 °C, 20 s	58 °C, 20 s	72 °C, 15–30 s kb^−1^
27			72 °C, 5 min
- To digest any template DNA, add 0.5 μL DpnI and incubate the reaction mixture at 37 °C for 2 h followed by a heat inactivation step at 80 °C for 20 min.
- Use 1–5 μL of the PCR product to transform the top ten competent cells. Select for colonies by plating the cells on LB agar containing the appropriate antibiotic for selection of the selected plasmid. Incubate plates at 37 °C for 16–18 h overnight.
- Set up overnights of several colonies, and isolate plasmid DNA from each using a plasmid isolation kit according to the manufacturer’s protocol. Verify correct incorporation of amber mutation through sequencing (e.g., Genewiz). Plasmid DNA is stable at −20 °C for at least a year.
  ◆ TROUBLESHOOTING

## Procedure 3: *E. coli* protein expression

● TIMING ~5–6 days

### Expression of ST and WT SC LASL constructs in *E. coli*

1. Transform chemically or electrically competent BL21(DE3) *E. coli* with the desired plasmid. Select for colonies by plating the cells on LB agar containing the corresponding antibiotic for the chosen plasmid. Incubate plates at 37 °C for 16–18 h overnight.
2. Inoculate a single colony into 2–5 mL LB medium containing the appropriate antibiotic and incubate overnight culture at 37 °C with shaking at 250 rpm.
3. Inoculate from the overnight culture at a 1:100 ratio into a large shaker flask containing LB medium and the corresponding antibiotic.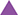 **CRITICAL STEP** For proper aeration, a 1:4 media to shaker flask volume is recommended.
4. Incubate culture with shaking (250 rpm) at 37 °C until culture reaches an optical density at *λ* = 600 nm of 0.6.
5. Induce protein expression with 0.5 mM IPTG and incubate overnight at 18 °C shaking at 180 rpm.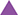 **CRITICAL STEP** Alternative conditions may be required for expression of various fusion proteins (e.g., temperature, inducer concentration, inoculation time).

### Expression of pSC LASL constructs in *E. coli*

- Cotransform 25 ng of the pSC expression vector along with 25 ng pEVOL–NBK-1 plasmid into chemically or electrically competent BL21(DE3) *E. coli*.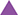 **CRITICAL STEP** Test a range of 25–100 ng of plasmid for transformation. If expression yields are low, the *E. coli* B95 host strain, which has no specific assignment of the UAG codon, can be alternatively used for expression.
  ◆ TROUBLESHHOOTING
- Plate dilutions of the cotransfected cells on LB agar plates containing chloramphenicol (30 μg mL^−1^) and the antibiotic corresponding to the resistance marker of the expression vector. Incubate selection plates at 37 °C overnight.
- Inoculate a single colony into 2–5 mL LB medium containing 30 μg mL^−1^ chloramphenicol and the antibiotic corresponding to the resistance marker of the expression vector. Incubate culture with agitation overnight (37 °C, 250 rpm).
- Inoculate overnight at a 1:50 ratio in a large shaker flask containing Terrific Broth medium and 30 μg mL^−1^ chloramphenicol and the antibiotic corresponding to the resistance marker of the expression vector.
- Incubate the culture with shaking (250 rpm at 37 °C) until *E. coli* reach an optical density at *λ* = 600 nm of 0.6. Add 500 μL of the pre-induction sample to a microcentrifuge tube, spin down, remove supernatant and store pellet at −20 °C.
- Weigh out Lys(*o*NB) necessary for a 1 mM final concentration in culture and dissolve in 2 equivalents of 0.5 M NaOH. For example, for a 125 mL expression volume, dissolve 42 mg Lys(*o*NB) in 500 μL 0.5 M NaOH.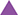 **CRITICAL STEP** Always prepare Lys(*o*NB) solution fresh and minimize exposure to light.
- Upon reaching an OD of 0.6, induce expression of pSC by adding IPTG (final concentration of 0.5 mM), expression of the aaRS/tRNA pair by adding arabinose (final concentration 0.1% (wt/vol)) and adding the Lys(*o*NB) solution from Step 11 (final concentration 1 mM).
- Shake flask at 200 rpm overnight at 18 °C.

### Protein purification through Ni-NTA affinity

- Pellet the cells by centrifugation at 4,000*g* for 20 min at 4 °C.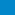 **PAUSE POINT** Pellets can be stored at −80 °C for several months before further purification.
- Resuspend the cell pellet in 40 mL of lysis buffer containing complete EDTA-free protease inhibitor cocktail tablets.
- Sonicate the cell suspension on ice (6× 3 min cycles at 30% amplitude, 33% duty cycle, 1 s on 2 s off, with 3 min rest between each cycle). Add 500 μL of the lysed cell suspension to a microcentrifuge tube and store at −20 °C.
- Centrifuge the sonicated cell suspension at 5,000*g* for 20 min at 4 °C. Add 500 μL of the clarified lysate to a microcentrifuge tube and store at −20 °C.
- Apply the clarified lysate supernatant to a column containing Ni-NTA resin equilibrated with lysis buffer.
- Wash the column (2 CV, 6×) with the wash buffer.
- Elute the His-tag bound protein with 5–10 CV of elution buffer, collecting each 1 mL elution in a separate microcentrifuge tube.
- Measure absorbance of the collected elution fractions at 280 nm using a spectrophotometer to determine protein concentration. Use the elution buffer as the blank.
  ◆ TROUBLESHOOTING

### Protein characterization through SDS–PAGE and MS

- Prepare pre-induction, postinduction, soluble, insoluble and elution samples for SDS–PAGE by mixing 50 μL of each with 50 μL of 2× Laemelli sample buffer.
- Heat the protein samples at 95 °C for ~10 min and centrifuge samples for 2 min at 1,000*g*.
- Set up an 8–16% TGX mini-Protean precast gradient gel in the electrophoresis apparatus, add SDS–PAGE running buffer, load the samples (10 μL each) and carry out the electrophoretic separation at 120 V for 60 min. Include a molecular weight standard to estimate the mass of sample proteins.
- Stain the proteins in the gel with Brilliant Blue R or a suitable alternative and image using a gel imaging system.
- For mass spectrometry, remove imidazole from the protein elution fractions using a Zeba desalting column and add 40 μL of the desalted protein to a mass spectrometry vial at a concentration of 0.5 mg mL^−1^. Analyze the protein sample under positive mode, using a water:acetonitrile gradient containing 0.1% FA.
- On the basis of characterization, pool desired elution fractions, add 30% (vol/vol) glycerol and store at −20 °C.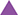 **CRITICAL STEP** The stability of protein will vary but can be often stored for 2–12 months. Minimize freeze–thaw cycles. Glycerol concentration can range from 10% to 50%.
  ◆ TROUBLESHOOTING

## Procedure 4: biomaterial incorporation

● TIMING <9 days

### Expression of LASL constructs for biomaterial incorporation

1. SC–*x*POI–LPETG, ST, pSC–*x*POI-LPETG and SrtA_7M_ protein stock solutions. Generate the SC–*x*POI–LPETG, ST and pSC–*x*POI–LPETG plasmids for biomaterial incorporation as described in Procedure 2. Express SC–*x*POI–LPETG, ST, pSC–*x*POI–LPETG and SrtA_7M_ in *E. coli* according to the procedure in Procedure 3 (Steps 1–5 for SC and ST, and SrtA_7M_ expression, and Steps 6–13 for pSC expression). Purify and characterize the purified proteins according to Steps 14–27 in Procedure 3. Store protein stock solutions at −20 °C.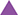 **CRITICAL STEP** Stability of protein will vary but can be often stored for 2–12 months. Minimize freeze–thaw cycles. Glycerol concentration can range from 10% to 50%.

### Sortase functionalization

- Mix pSC–*x*POI–LPETG, SrtA7M and H-GGGGDDK(N_3_)-NH_2_ peptide at the molar ratios indicated in the table below in a microcentrifuge tube. The total reaction volume should be 1 mL. Incubate the mixture for 1.5 h at 37 °C. The H-GGGGDDK(N_3_)-NH_2_ peptide synthesis is described in Box 3 (also see Table 6).

**Table:** 

Component	Molecular weight (g mol^−1^)	Molar ratio
pSC–*x*POI–LPETG	Variable	1
SrtA7M	17,851.14	0.1
H-GGGGDDK(N3)-NH2	900	100
STEPL Buffer	–	–
- To a microcentrifuge tube containing 100 μL of Ni-NTA resin, add 1 mL lysis buffer. Centrifuge at 500*g* for 1 min and remove the supernatant. Repeat this step two times.
- Apply the 1 mL reaction mixture from Step 2 to the Ni-NTA resin and shake for 1 h. Following incubation, centrifuge the tube at 500*g* for 1 min.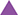 **CRITICAL STEP** SrtA_7M_ and unreacted pSC–*x*POI–LPETG will remain attached to the Ni-NTA resin, while the pSC–*x*POI–N_3_ will be in the supernatant.
- To remove excess peptide and generate pSC–*x*POI–N_3_, apply the supernatant from Step 4 to a Zeba desalting column pre-equilibrated with PBS according to the manufacturer’s protocol.
- Analyze the protein sample with SDS–PAGE and mass spectrometry to determine the extent of functionalization according to Procedure 3, Steps 22–27.
  ◆ TROUBLESHOOTING

### Incorporation into biomaterials

- Treat two glass slides with Rain-X and place 0.5 mm silicone spacers on either end of one coated slide.
- To prepare a 5 μL gel functionalized with pSC–*x*POI–N_3_ at a concentration of 12 μM, add pSC–*x*POI–N_3_ and PEG-tetraBCN solution, and PBS to a microcentrifuge tube and incubate for 1 h. Then, add PEG-diazide and gently mix. The synthesis and preparation of PEG-tetraBCN and PEG-diazide are described in Box 2. The table below provides an example of stock solution concentrations, final concentrations and volumes:

**Table:** 

Component	Stock concentration (mM)	Final concentration (mM)	Vol (μL)
pSC–*x*POI–N_3_	0.05	0.012	1.23
PEG-tetraBCN	10	4	2
PEG-diazide	40	8	1
Buffer	–	–	0.77
Total volume			5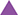 **CRITICAL STEP** Upon addition of the PEG-diazide, the solution will begin to gel therefore transfer to the glass slides should be rapid. The initial properties of the gel, such as the cross-linking density and concentration of the pSC–*x*POI–N_3_ can be tuned by adjusting the initial concentration of macromer and protein added.
  ◆ TROUBLESHOOTING
- Using a positive displacement pipette, aliquot 5 μL of the gel-precursor solution onto the Rain-X-treated glass slide with the 0.5 mm rubber spacers and carefully cover the solution by placing the second glass slide on top. Allow network formation to proceed for 45 min at room temperature.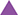 **CRITICAL STEP** The size and thickness of the gel can be adjusted by changing the volume of solution aliquoted and size of the rubber spacers. Various molds can be used to change the shape of the gel as well.
- Upon gelation, gently remove the top slide and place the formed gel into a well containing excess PBS buffer (5–10 mL) to allow unreacted PEG and protein to diffuse out.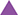 **CRITICAL STEP** For homogeneously functionalized gels, let the gel wash for a minimum of ~12 h and exchange PBS buffer twice throughout the washing step. The gel can be incubated at room temperature or 4 °C, depending on the temperature sensitivity of the protein and gel components.

### Hydrogel patterning

- Spatiotemporal hydrogel patterning can be achieved through (1) flood illumination, (2) single photon mask-based photopatterning or (3) two-photon lithography.
  - Flood illumination
    - Set up a 365 nm light source (e.g., OmniCure) further outfitted with a 365 nm band-pass filter between the light source and sample. Tune the intensity of the UV light between 5 and 20 mW cm^−2^ at the sample height using a radiometer. When taking measurements, make sure the radiometer sensor is at the center of the light source. Adjust the collimating lens such that the light intensity is homogeneous over the desired area.
    - Place a pSC–*x*POI–N_3_-functionalized gel on a glass slide, cover the gel with PBS buffer to prevent the gel from drying and place the sample at the center of the light source.
    - Turn the light source on and irradiate the sample.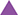 **CRITICAL STEP** The light dosage determines the amount of uncaging and active SC. Users should tune the light intensity and the length of exposure based on the desired extent of uncaging.
  - Mask-based patterning
    - Repeat the setup of the light source and hydrogel in Step 11A(i,ii), except place 0.5 mm rubber spacers on either side of gel to support a photomask and place the desired pattern on the gel. Verify that the gel is directly below the center of the light source.
      ◆ TROUBLESHOOTING
    - Turn the light source on and irradiate the sample to pattern activation of pSC.
  - Two-photon lithography
    - Incubate a pSC–*x*POI–N_3_-functionalized hydrogel in 3 mL of 50 μM Rhodamine B solution for a minimum of 4 h.
    - Place the gel at the center of the 2P patterning chamber, coat the rubber with clear nail polish and apply a second glass slide on top. The chamber should be completely sealed.
    - Secure the chamber with the hydrogel in a 100 mm dish, fill the dish with PBS and place it on the stage of the multiphoton microscope. Find the surface of the gel by imaging the first plane that fluoresces with Rhodamine B or by looking for dust particles or surface aberrations.
    - 3D images or designs of interest can be generated using the software Autodesk.
    - Irradiate the samples by scanning regions of interest with a 740 nm laser at 100% laser power using 30 scan repeats with a pixel dwell time of 3,200 ns, and a *z*-spacing of 2 μm for each image slice.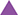 **CRITICAL STEP** Out-of-plane irradiation from the laser will cause a loss in the resolution of the pattern. The *z*-spacing of each image slice can be tuned.
      ◆ TROUBLESHOOTING
- Upon completion of patterning, soak the exposed gel in a solution of excess ST protein for a minimum of 8 h.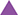 **CRITICAL STEP** The hydrogel can be soaked in excess ST protein before patterning. Following light exposure, excess ST and/or Rhodamine B can be removed by washing the gel in PBS overnight (~5 mL PBS buffer). For homogeneously functionalized gels, let the gel wash for a minimum of ~12 h and exchange the PBS buffer.

## Procedure 5: mammalian cell culture and characterization

● TIMING ~4–6 days

### Cell culture

1. For HEK293T cells, follow the vendor culturing protocol. In brief, grow HEK293T cells in growth media in a T25 flask maintained at 37 °C and 5% CO_2_.
2. Passage HEK293T cells when they reach 70% confluency. Remove the growth medium, wash the cells with 5 mL of PBS and add 2 mL of 0.25% trypsin and incubate cells at room temperature for 5 min. Add 5 mL of medium to inactivate trypsin, pipette up and down to detach cells from the flask, transfer the cell suspension to a 50 mL tube and centrifuge at 200*g* for 5 min.
3. Resuspend the cell pellet in 1 mL of growth medium and count the cells. Seed cells in a new T25 flask for culturing or seed cells for transfection.
4. For transfection experiments, coat 35 mm glass-bottom dishes with 100 μL of prewarmed 0.1% gelatin.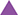 **CRITICAL STEP** Improved HEK293T cell adherence was observed upon gelatin addition to the glass dish but may not be necessary for other cell types.
5. Seed each well with ~50,000 HEK293T cells cm^−2^ in 250 μL of media.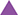 **CRITICAL STEP** Homogeneous seeding of cells is critical for optimal transfection efficiency. Seeding densities will vary based on the cell line (e.g., ~2,500 cells cm^−2^ for mouse dermal fibroblast).
6. Incubate the cells at 37 °C and 5% CO_2_ overnight.

### WT SC transfection

- For transfection of the WT LASL plasmid, mix the following components per well to be transfected in a microcentrifuge tube:

**Table:** 

Component	Amount per well
SC plasmid	1 μ9
Opti-MEM	35 μL
  ◆ TROUBLESHOOTING
- Mix 35 μL of Opti-MEM buffer and 1 μL of lipofectamine reagent per well to be transfected in a separate microcentrifuge tube.
- Combine the DNA and lipofectamine solutions and incubate at room temperature for 30 min.
- Mix the transfection mixture in 250 μL of growth media per well to be transfected.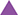 **CRITICAL STEP** The media containing the transfection reagents should be gently but thoroughly mixed for homogeneous transfection of cells.
- Retrieve the confluent seeded cells from Step 6, remove the media in the well, and gently add the new transfection media from Step 10 to the cells.
- Transfer the cells to an incubator at 37 °C, 5% CO_2_ and incubate for 24–72 h after transfection.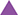 **CRITICAL STEP** The incubation period with the transfection reagent will vary and depend on the construct, seeding density and transfection process.

### pSC transfection

- For transfection of the photocaged LASL and pNeu–hMbPylRS–4xU6M15 expression plasmids, mix the following components in a microcentrifuge tube:

**Table:** 

Component	Amount per well
pSC plasmid	0.5 μg
pNeu–hMbPylRS–4xU6M15	0.5 μ9
Opti-MEM	35 μL
- Mix 35 μL of Opti-MEM buffer and 1 μL of lipofectamine 3000 reagent per well to be transfected in a separate microcentrifuge tube.
- Combine the DNA and lipid solutions and incubate at room temperature for 30 min.
- Mix the transfection mixture with 250 μL of 2.5 mM Lys(*o*NB) growth media (see the ‘Reagent setup’ for Procedure 5) per well to be transfected.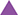 **CRITICAL STEP** The mixture should be gently but thoroughly mixed for homogeneous transfection of cells.
  ◆ TROUBLESHOOTING
- Remove old media from seeded wells, and gently add new transfection media.
- Transfer the cells to an incubator at 37 °C, 5% CO_2_ for 24–72 h after lipid transfection for optimal expression of the LASL construct.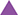 **CRITICAL STEP** Expression times will vary depending on construct, seeding density and transfection process.

### Light exposure

- Before light exposure, remove media from transfected cells and wash cells by gently adding 100 μL of warm HBSS then replacing with 100 μL of fresh HBSS.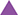 **CRITICAL STEP** Carefully wash cells to avoid cell detachment. If the cell line is sensitive to long periods in HBSS, media lacking phenol red can be used during light exposure experiments.
- Spatiotemporal patterning can be achieved through (1) flood illumination or (2) single-photon mask-based photopatterning:
  - In vitro activation through flood illumination
    - Set up a 365 nm light source, such as an OmniCure, and place a 365 nm glass filter above it. Tune the intensity of the UV light to 5–20 mW cm^−2^ at the sample height using a radiometer. When taking measurements, make sure the radiometer sensor is at the center of the light source. Adjust the collimating lens such that the light intensity is homogeneous over the desired area.
    - Place the transfected cell plate at the center of the light.
    - Turn the light source on and irradiate the sample.
  - In vitro patterning through mask-based single photon activation
    - Repeat Step 20A(i and ii) setup of the light source and 35 mm cell plate. For patterning, a rough opaque mask of any desired shape can be placed on the glass filter or replaced with a chrome photomask containing the desired patterns. Verify that the plate is directly above the center of the light source.
    - Turn the light source on and irradiate the sample to pattern activation of pSC.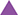 **CRITICAL STEP** Photoactivation experiments should ideally be carried out in sterile conditions. Exposures can be conducted at 37 °C, 5% CO_2_ by using a live-cell imaging system. During light exposure, place a sterile black rubber cover on top of the well to reduce light scattering from the plastic lid. Use a glass well plate to avoid exposure through plastic, which will result in light scattering. The light dosage determines the amount of uncaging and active SC. Users should tune the light intensity and the length of exposure based on desired uncaging. Complete uncaging of intracellular pSC was seen <15 min at 20 mW cm^−2^. For more sensitive cell lines, light exposure can be conducted at 5 mW cm^−2^.
      ◆ TROUBLESHOOTING
- Following light exposure, replace HBSS with 250 μL of growth medium without Lys(*o*NB) in each well and incubate cells at 37 °C and 5% CO_2_ until downstream analysis.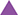 **CRITICAL STEP** The incubation period before downstream analysis will vary depending on constructs, biological response and kinetics. It is recommended that cells be monitored for 0–72 h and an optimal end point selected. Although SC/ST ligation was observed in <30 min following light exposure for in-solution experiments, longer incubation periods may allow for more complete ST/SC ligation.
  ◆ TROUBLESHOOTING

## Troubleshooting

Troubleshooting advice can be found in Table 7.

## Timing

Procedure 1, Steps 1–16, Lys(*o*NB) synthesis: 5 days

Procedure 2, Steps 1–14, Split-protein design: 1–4 days

Procedure 3, Steps 1–27, *E. coli* protein expression: 5–6 days

Procedure 4, Steps 1–12, Biomaterial incorporation: 9 days

Procedure 5, Steps 1–21, Mammalian cell culture and characterization: 4–6 days

Box 2, Synthesis of gel precursors: 3 days

Box 3, Synthesis of the H-GGGGDDK(N_3_)-NH_2_ polyglycine probe: 4 days

## Anticipated results

Expected results using example proteins for LASL in solution (Fig. 2), biomaterials (Box 1) and in vitro (Fig. 8) are shown.

### Lys(*o*NB) (for chemical structure, see Fig. 5)

^1^H NMR (500 MHz, DMSO d6): δ 8.10 (dd, *J* = 8.2, 1.3 Hz, 1H), 7.81 (td, *J* = 7.6, 1.3 Hz, 1H), 7.65 (dd, *J* = 7.9, 1.4 Hz, 1H), 7.60 (td, *J* = 7.7, 1.4 Hz, 1H), 7.45 (t, *J* = 5.7 Hz, 1H), 5.35 (s, 2H), 3.50 (d, *J* = 6.2 Hz, 1H), 2.99 (q, *J* = 6.5 Hz, 2H), 1.81–1.63 (m, 2H), 1.45–1.28 (m, 4H). Spectra calibrated to residual solvent. MS (LTQ–MS): calculated for C_14_H_20_N_3_O_6_ ([M+H]^+^): 326.1; found, 326.1. These spectral data matched those previously reported^18^.

### PEG-tetraBCN (for chemical structure, see Box 2)

White solid, ^1^H NMR (500 MHz, CDCl3) δ 5.29 (s, 4H), 4.14 (t, *J* = 13.1 Hz, 8H), 3.78 (dd, *J* = 11.5, 6.2 Hz, 16H), 3.65 (s, 1824H), 3.52–3.49 (m, 8H), 3.42 (s, 8H), 3.38 (t, *J* = 4.9 Hz, 8H), 2.75 (s, 16H), 2.37–2.13 (m, 16H) 1.66–1.51 (m, 8H), 1.42–1.33 (m, 4H), 1.02–0.90 (m, 8H). Spectra calibrated to residual solvent. Functionalization was confirmed to be >95% by ^1^H NMR by comparing integral values for characteristic BCN peaks (δ 2.24, 1.57, 1.34, 0.92) with those from the PEG pentaerythritol core (δ 3.40–3.45).

### PEG-diazide (for chemical structure, see Box 2)

White solid, ^1^H NMR (500 MHz, CDCl_3_) δ 3.66–3.61 (m, 182H), 3.38 (t, *J* = 13.1 Hz, 4H). Spectra calibrated to residual solvent. Functionalization was found to be >90% by comparing integral values for hydrogens introduced upon azide coupling (δ 3.38) with those from the PEG (δ 3.65).

### H-GGGGDDK(N_3_)-NH_2_ polyglycine peptide (for chemical structure, see Box 3)

White solid, LC–MS (electrospray ionization): calculated for C_26_H_43_N_12_O_12_^+^ [M + ^1^H]^+^, 715.31; observed 715.40.

## Supplementary Material

1

## Figures and Tables

**Table 1 |:** Amino acid sequence of ST and SC

ST	AHIVMVDAYKPTK
SC	MVDTLSGLSSEQGQSGDMTIEEDSATHIKFS**K**RDEDGKELAGATME LRDSSGKTISTWISDGQVKDFYLYPGKYTFVETAAPDGYEVATAITFTVNEQGQVTVNGKATKGDAHI

**Table 2 |:** Examples of plasmids for bacterial expression of LASL components

Construct insert	Promoter	Replication	Purification Tag	Vector, resistance	Linker
nPOI–linker–ST–6×His	T7	P15A	6×His	pET-29a (kanamycin)	GGSGGGGSGGS
SC–linker–cPOI–6×His or SC–linker–cPOI–LPETG–6×His	T7	P15A	6×His	pET-21a (carbenicillin)	GGSGGGSGG
(MmPylRS; Y306M, L309A, C348A, Y384F) x2–Pyl–tRNACUA	araBAD, glnS’	P15A	–	pEVOL (chloramphenicol)	–

**Table 3 |:** Examples of plasmids for mammalian expression of LASL components

Construct insert examples	Promoter	Vector, resistance	Linker, P2A AA sequence
SC–linker1–cPOI–P2A–nPOI–linker2–ST–P2A–mCh	CMV	pcDNA3.1 (carbenicillin, HygR)	Linker 1: GGSGGGSGG Linker 2: GGSGGGSGGGSG P2A: GSGATNFSLLKQAGDVEENPGP
nPOI–ST–P2A–SC–cPOI	CMV	pcDNA3.1 (carbenicillin, HygR)	
POI–linker1–ST–P2A–SC–Iinker2–CAAX	CMV	pcDNA3.1 (carbenicillin, HygR)	Linker 1: GGSGGGSGGGSG Linker 2: GGSGGGSGG P2A: GSGATNFSLLKQAGDVEENPGP CAAX: MSKDGKKKKKKSKTKCVIM
hMbPylRS(Y271A, Y349F)–4x(M15-tRNACUA)	CMV, U6	pcDNA3.1 (carbenicillin, NeoR)	—

**Table 4 |:** Codon-optimized sequences of SC/ST

ST	T7 vector: gcacacatagtaatggtagacgcctacaagccgacgaag T5 vector: gctcatatcgtcatggttgacgcgtataaaccgaccaaa
SC	atggttgataccttatcaggtttatcaagtgagcaaggtcagtccggtgatatgacaattgaagaa gatagtgctacccatattaaattctca**AAA**cgtgatgaggacggcaaagagttagctggtgca actatggagttgcgtgattcatctggtaaaactattagtacatggatttcagatggacaagtgaaag atttctacctgtatccaggaaaatatacatttgtcgaaaccgcagcaccagacggttatgaggtag caactgctattacctttacagttaatgagcaaggtcaggttactgtaaatggcaaagcaactaaag gtgacgctcatatt

**Table 5 |:** Primers to install amber stop codon (TAG) at SC’s Lys(31)

Primer name	Sequence 5’→3’
SpyCatcher-SDM-Forward	attctcatagcgtgatgaggacggcaaagagttag
SpyCatcher-SDM-Reverse	catcacgctatgagaatttaatatgggtagcactatcttc

**Table 6 |:** Peptide synthesis protocol of Boc-GGGGDDK(Dde)-NH_2_

Step	Operation	Parameter	Volume (mL)	Drain	Power (Watts)	Temperature	Time
1	Wash	DMF	10	Yes			
2	Add capping		10	No	40	65	30 s on, 30 s off for 2 min
3	Microwave method	Initial deprotection		Yes	45	75	30 s
4	Wash		20	Yes			
5	Add capping		10	No			
6	Microwave method	Deprotection		Yes	45	75	180 s
7	Wash–top	DMF	10	Yes			
8	Wash–bottom	DMF	10	Yes			
9	Wash–top	DMF	10	Yes			
10	Add amino acid		10	No			
11	Add activator		4	No			
12	Add activator base		2	No			
13	Microwave method	Coupling		Yes	45	75	300 s
14	Wash–top	DMF	10	Yes			
15	Wash–bottom	DMF	10	Yes			

**Table 7 |:** Troubleshooting table

Step	Problem	Possible reason	Solution
**Procedure 1**			
1	Poor reaction yield	H_2_O present in reagents	Verify all reagents are carefully stored to prevent moisture
		H_2_O present in flask	Flame dry round-bottom flask
9	Poor separation	Crude product volume	Resuspend crude product in minimal solvent to maximize product separation and purity
**Procedure 2**			
14	Incorrect or no successful mutation	Degraded or expired antibiotic in plates	Generate fresh agar plates
		WT plasmid present	Verify Dpnl enzyme is properly working
		High SC DNA template present	Reduce intial SC DNA template to 10 ng
**Procedure 3**			
6	No colonies	DNA concentration	Test a range of plasmid concentrations
21	Poor protein yield	Insoluble protein	Purify through denaturing conditions
		Protein sequence design	Redesign protein with solubility tags (see Procedure 2)
		Protein degradation	Add additional protease cocktail inhibitors
		Temperature conditions	Test a variety of temperatures for protein expression
27	Protein crashing out	High number of freeze–thaw cycles	Aliquot protein to minimize freeze–thaw cycles
		Poor protein stability	Increase glycerol concentration
**Procedure 4**			
6	pSC–*x*POI–LPETG present in elution	High Ni-NTA present in pSC–*x*POI–LPETG protein stock	Desalt pSC–*x*POI–LPETG before sortase functionalization
	Low or no incorporation of reactive handle on POI	Sortase enzyme degraded	Use a fresh batch of SrtA_7M_ enzyme
		pH	Ensure pH is ~7–8 before beginning sortagging reaction
8	Poor gelation	Incorrect concentrations	Verify concentrations of all stock solutions
	Poor incorporation of pSC–*x*POI–N_3_	pSC–*x*POI–N_3_ stock contains SrtA_7M_	Add more Ni-NTA resin during reverse affinity purification of pSC to remove excess sortase
11B(i)	Poor patterning	Photomask placed directly on gel without spacers	Add spacers to support photomask and prevent distortion of gel during patterning
11C	Poor patterning	Gel distorted	Avoid compressing the gel during patterning
		Gel placed at an angle	Verify that gel is on a flat surface to facilitate patterning on an even *z* plane
**Procedure 5**			
7, 16	Cell death	Low seeding density	Seed cells at a higher concentration to reach 70–80% confluency during transfection
		High seeding density	Seed cells at a lower concentration to prevent detachment of cells in a sheet-like layer
		High transfection reagent concentration	Test a range of transfection concentrations to optimize cell viability
	Low protein expression	Plasmid design	Revisit Procedure 2
20	Cell death	Excessive exposure to 365 nm light	Reduce length or intensity of light exposure
		Media not swapped to PBS	Incubate cells in PBS or media without phenol red
21	Poor SC/ST ligation following light exposure	Plasmid design	Verify proper ligation with WT construct, redesign construct (see Procedure 2)
		Incubation period	Test a range of incubation times to maximize SpyLigation

## Data Availability

All pertinent experimental and characterization data discussed in this protocol are available in the supporting primary research articles^11,26^ and within this paper. Plasmids generated during the study are available on Addgene or from the corresponding author upon reasonable request.

## Box 1 Photolithographic patterning techniques

### Flood illumination:

in flood illumination, the entire sample is exposed to light. While this technique does not provide spatial control, the dosage of light the sample receives can be controlled by tuning the intensity of the light source as well as the exposure time to dictate the extent of response. In this protocol, we use a 365 nm light source and typically use intensities between 0 and 20 mW cm^−2^.

### Mask-based patterning:

in this technique, a photomask that partially obstructs light in user-defined two-dimensional patterns is placed between the substrate and the light source, afording *x*–*y* control over spatial patterning with resolution on the order of tens of microns. This technique is inexpensive and simple photomasks can be constructed out of readily available materials (e.g., rubber). To create more complex patterns, chrome-coated glass photomasks can be designed and ordered from commercial suppliers.

### Two-photon lithography:

in multiphoton laser-scanning lithographic patterning, a femtosecond-pulsed laser is directed to a confined focal point with submicrometer control over the *x* and *y* dimension and 2–3 μm in the *z* dimension. By raster scanning the laser focal point while tuning the laser intensity, light can be used to pattern complex activation of photoresponsive chemistries. To generate 3D patterns, a 3D structure can be computationally processed into a stack of two-dimensional slices that can be raster scanned through a photoresponsive material by stepping through *z*-dimensional defined sections. In this protocol, we used a 740 nm pulsed near-infrared laser.

## Box 2 Synthesis of gel precursors

● Timing <3 days

### Additional reagents

- Four-arm PEG tetraamine (M_n_ ~10,000 Da, JenKem, cat. no. A7011–1) (1*R*,8S,9s)-Bicyclo[6.1.0]non-4-yn-9-ylmethyl *N*-succinimidyl carbonate (endo) (BCN-OSu) (Sigma, cat. no. 744867–100MG)
   **CRITICAL STEP** Alternatively, BCN-OSu can be synthesized as previously described^60^.
- SpectraPor 7 dialysis tubing, MWCO 2 kDa (Repligen, cat. no. 132109)
- PEG 2000 (M_n_ ~2,000 Da, PEG 2000) (Sigma, cat. no. 8210371000)
- Methanesulfonyl chloride (MsCl) (Sigma, cat. no. 471259–5ML)
- Sodium azide (NaN_3_) (Thermofisher, cat. no. S227I-100)
   **CAUTION** NaN_3_ is fatal if swallowed, in contact with skin or inhaled.
- Pyridine anhydrous (Sigma, cat. no. 270970–100ML)
   **CAUTION** Pyridine is highly flammable, acutely harmful upon inhalation or contact with skin and causes eye irritation.
- Magnesium sulfate anhydrous (Thermofisher, cat. no. M653)
- Sodium bicarbonate (NaHCO_3_) (Thermofisher, cat. no. S233–500)
- Celite (Sigma, cat. no. 1026930250)
- Deuterated chloroform (CDCl_3_) (Sigma, cat. no. 225789–100G)

### Reagent setup

#### Saturated sodium bicarbonate

Dissolve 8 g of NaHCO_3_ in 100 mL of DI water. Filter to remove any undissolved solids.

### Procedure

#### Synthesis of 10 kDa PEG-tetraBCN

1. In a 50 mL dry round-bottom flask with a stir bar, add 1.143 g of PEG-10k NH_2_Cl (0.050 mmol PEG, 0.2 mmol total NH_2_, 1 equiv.) and 100 mg of BCN-OSu (0.343 mmol, 1.5 equiv. of BCN-OSu relative to total NH_2_).
2. Measure out 5 mL of dried DMF under N2 gas and add to the round-bottom flask.
3. Add 159 μL DIEA (0.8 mmol, 4 equiv.) to the flask and stir overnight at room temperature.
4. Following the overnight reaction, add 50 mL of deionized (DI) H_2_O to the flask.
   **CRITICAL STEP** Addition of water is to prevent the DMF from dissolving the dialysis tubing.
5. Rinse the SpectraPor dialysis tubing in DI H_2_O before beginning dialysis, as it is stored in dilute NaN3 which will react with the BCN.
6. Pour the reaction mixture into the tubing, and dialyze against DI H_2_O (2 L) at 4 °C. Change dialysis water at 2.5 and 5 h, then dialyze overnight.
7. Pour the PEG-tetraBCN solution from the dialysis membrane into 50 mL conical tubes and snap freeze in liquid nitrogen.
8. Lyophilize for 3 days to yield an of-white powder (1.06 g, quantitative yield).
9. Characterize the product using ^1^H NMR. Weigh 5 mg of PEG-tetraBCN and dissolve in 500 μL of CDCl_3_. Transfer the solution into an NMR tube and obtain NMR data of the compound. Use chemical shifts and integration of peaks to confirm the chemical structure. Confirm functionalization by comparing the integral values for characteristic BCN peaks with those from the PEG backbone.
10. Weigh out PEG-tetraBCN and dissolve in PBS to give a final concentration of 10 mM. Vortex the solution thoroughly and allow it to hydrate for 15 min. Aliquot 100 μL into microcentrifuge tubes and store at −80 °C.
   **CRITICAL STEP** Assume the denisty of PEG-tetraBCN is 1 g mL^−1^ and subtract the polymer volume from the final volume of PBS calculated. PEG-tetraBCN is stable for up to a year if properly stored. Minimize freeze–thaw cycles and exposure to warm temperature, light and oxygen.

#### Synthesis of 2 kDa PEG-diazide

1. In a 50 mL dry round-bottom flask with a stir bar, combine 5 g of 2 kDa linear PEG 2000 (5 mmol total OH, 1 equiv.) and purge the flask with argon.
2. To the 50 mL flask, add 40 mL of dry DCM and 10 mL of anhydrous pyridine. Submerge the flask in an ice bath held at 0 °C.
3. Add 1.935 mL of MsCl (25 mmol, 5 equiv.) to the mixture, and stir the reaction overnight.
4. Concentrate the mixture under reduced pressure with a rotary evaporator.
5. Wash the reaction with 100 mL of saturated sodium bicarbonate (3×).
6. Wash the organic layer with saturated Na_2_SO_4_. Filter the organic layer to remove any solids using a Buchner funnel setup. Distill the crude material under reduced pressure with the rotary evaporator.
7. Precipitate the concentrated product from step 6 above by adding 15 mL of cold diethyl ether to the flask three times.
8. Transfer the solid to a 50 mL conical tube, and spin down at 4,000*g* for 5 min. Pipette off excess diethyl ether and dry the solid by blowing nitrogen over the product. Lyophilize the product overnight to remove remaining solvent and obtain the intermediate activated mesylate PEG product (~5 g, quantitative yield).
9. In a 50 mL dry round-bottom flask with a stir bar, combine 5 g mesylate intermediate (5 mmol total mesyl groups), 1.625 g of sodium azide (25 mmol, 5×) and 10 mL of anhydrous DMF.
10. Purge the reaction with argon.
11. Heat flask to 80 °C and run the reaction overnight under argon.
12. Filter the reaction through Celite and concentrate the material under reduced pressure with the rotary evaporator.
13. Dilute the reaction in 15 mL of water and dialyze against water using 1 kDa SpectraPor tubing. Change water at 2.5 and 5 h then dialyze overnight.
14. Pour the PEG-diazide from the dialysis membrane into 50 mL conical tubes and snap freeze in liquid nitrogen.
15. Lyophilize for 3 days to yield a white powder (5.00 g, quantitative yield).
16. Characterize the product using ^1^H NMR. Weigh 5 mg of PEG-diazide and dissolve in 500 μL of CDCl_3_. Transfer solution into an NMR tube and obtain NMR data of compound. Use chemical shifts and integration of peaks to confirm the chemical structure.
17. Weigh out PEG-diazide and dissolve in PBS at a final concentration of 40 mM. Vortex the PEG-diazide solution thoroughly, and allow it to hydrate for 15 min. Aliquot 100 μL into microcentrifuge tubes and store at −80 °C.
   **CRITICAL STEP** Assume the PEG-diazide density is 1 g mL^−1^ and subtract the polymer volume from the final volume of PBS calculated. PEG is stable for up to a year if properly stored. Minimize freeze–thaw cycles and exposure to warm temperature, light and oxygen.

## Box 3 Synthesis of the H-GGGGDDK(N_3_)-NH_2_ polyglycine probe

● TIMING <4 days

This box describes the automated solid-phase peptide synthesis of the Boc-GGGGDDK(Dde)-NH2 and functionalization with to generate the H-GGGGDDK(N3)-NH2 polyglycine probe.

### Materials

#### Additional reagents

- 1-Hydroxybenzotriazole (Chem-Impex, cat. no. 24755)
   **CAUTION** 1-Hydroxybenzotriazole is flammable and causes eye irritation.
- Piperidine (Chem-Impex, cat. no. 02351–1L)
   **CAUTION** Piperidine is flammable and acutely corrosive and toxic upon inhalation or contact with skin or eyes.
- 2-(1H-benzotriazol-1-yl)-1,1,3,3-tetramethyluronium hexafluorophosphate (HBTU) (Chem-Impex, cat. no. 2011)
   **CAUTION** HBTU is harmful upon inhalation or contact with skin and causes eye irritation.
- DIEA (ChemImpex, cat. no. 00141)
   **CAUTION** DIEA is harmful upon inhalation or contact with skin and causes eye irritation.
- Trifluoroacetic acid (TFA) (Chem-Impex, cat. no. 2883)
   **CAUTION** TFA is acutely harmful upon inhalation or contact with skin and causes serious eye damage.
- 4-Azidobutanoic acid (Matrix Scientific, cat. no. 069400–1G)
- Hydrazine monohydrate (Sigma, cat. no. 207942)
   **CAUTION** Hydrazine is flammable and acutely corrosive and toxic upon inhalation or contact with skin and eyes.
- Rink-Amide Resin–ProTide (CEM, cat. no. R003-B5)
- Diethyl ether (ThermoFisher, cat. no. AC123990050)
   **CAUTION** Diethyl ether is flammable and harmful upon inhalation.
- Acetonitrile (ThermoFisher, cat. no. A9964)
   **CAUTION** Acetonitrile is acutely harmful upon inhalation or contact with skin and causes eye irritation.
- Acetic anhydride (Sigma, cat. no. 539996–25G)
   **CAUTION** Acetic anhydride is flammable, acutely toxic upon inhalation, corrosive upon contact with skin and causes severe eye damage.
- Standard Fmoc-protected amino acids
  - Boc-Gly-OH (Chem-Impex, cat. no. 00671)
  - Fmoc-Gly-OH (ChemPep, cat. no. 100801)
  - Fmoc-L-Asp(OtBu)-OH, (ChemPep, cat. no. 100402)
  - Fmoc-L-Lys-(Dde)-OH (Chem-Impex, cat. no. 03718)

#### Additional equipment

- Liberty 1 automated microwave peptide synthesizer (CEM)
- High-pressure liquid chromatograph (HPLC) (Dionex Ultimate 3000)
- HPLC column (ThermoFisher, 5 μm Synchronis silica 250 × 21.2 mm C18 column)

### Reagent setup

#### Fmoc–SPPS reagents

- Coupling reagent (0.5 M HBTU in DMF): weigh 7.59 g of HBTU and dissolve in 40 mL of DMF
- Base (2 M DIEA in DMF): mix 8.7 mL of DIEA with 25 mL DMF
- Deprotection solution (20% (vol/vol) piperidine in DMF): add 37.5 mL of piperidine to 150 mL DMF and mix thoroughly
- Capping solution: mix 20 mL DMF, 1 mL acetic anhydride and 140 μL of DIEA

#### Additional reagents

- 2% Hydrazine in DMF: mix 1.2 mL hydrazine with 58.8 mL DMF
- 95:2.5:2.5 (vol/vol) TFA:TIS:H_2_O: Mix 19 mL TFA and 0.5 mL of TIS with 0.5 mL H_2_O

### Procedure

1. In separate 50 mL centrifuge tubes, weigh Boc-Gly-OH, Fmoc-Gly-OH, Fmoc-Asp-(OtBu) and Fmoc-Lys-(Dde)-OH. Afterward, add DMF to dissolve each amino acid. The amounts for a 0.5 mmol reaction of Boc-GGGGDDK(Dde)-NH_2_ are listed in the table below.
  **Table:**
  Amino acid	Mass (g)	DMF vol (mL)
  Boc-Gly-OH	0.42	6
  Fmoc-Gly-OH	2.74	18
  Fmoc-Asp-(OtBu)	1.98	12
  Fmoc-Lys-(Dde)-OH	1.12	6
2. For the solid support, weigh 0.628 g Fmoc-Rink Amide resin (0.5 mmol, 1 equiv.) in a 15 mL tube.
3. Add 10 mL of DMF to the resin, vortex and allow to swell for 10 min.
4. Place the amino acids, coupling reagent, base and deprotection solutions on the CEM peptide synthesizer at their respective positions and add the resin to the reaction vessel.
5. Fill the DMF solvent container and open the supply line for the peptide synthesizer’s inert gas.
6. Start the synthesis protocol for Boc-GGGGDDK(Dde)-NH_2_. The peptide should be synthesized using the method in Table 6.
7. Following peptide synthesis, transfer the resin to a new glass vial, and wash the resin three times with 5 mL DMF.
8. To remove the 1-(4,4-dimethyl-2,6-dioxacyclohexylidene)ethyl (Dde) protecting group from the lysine, wash the resin three times for 10 min with 20 mL 2% hydrazine solution in DMF.
9. Remove the cleavage solution and wash the resin three times with 5 mL DMF.
10. For HATU coupling of the 4-azidobutanoic acid to the peptide, add 750 mg of HATU (1.97 mmol, 3.95 equiv. to NH_2_,), 227 μL of 4-azidobutanoic acid (2 mmol, 4 equiv. to NH_2_) and 1.38 mL DIEA (8 mmol, 16 equiv. to NH_2_) in minimal DMF to the resin in step 9 above and react for 1 hour.
11. Remove the solution from step 10 above and wash the resin three times in 5 mL DCM.
12. For peptide cleavage/deprotection, add 20 mL of 95:2.5:2.5 TFA:TIS:H_2_O solution and incubate the reaction for 3 h at room temperature.
13. Remove the liquid from the resin via filtration.
14. Precipitate the crude peptide by adding 180 mL of cold diethyl ether to the reaction in step 13 above and collect in 50 mL tubes.
15. Centrifuge tubes at 2,000*g* for 5 min at room temperature, decant the solvent and keep the precipitate.
16. Repeat steps 14–15 above for a total of two precipitation extractions.
17. Air dry the crude peptide samples and store at 4 °C until further analysis.
   **PAUSE POINT** Crude peptide can be stored at 4 °C for up to 2 months.
18. Perform semi-preparative reversed-phase HPLC with a C18 column to purify the products using a gradient from 5% to 100% acetonitrile:H_2_O containing TFA (0.1%) for 55 min, and a flowrate of 15 mL h^−1^. Collect the fractions of the main peak (~15 min) and characterize by direct inject mass spectrometry (positive mode).
19. Lyophilize the solutions containing the desired H-GGGGDDK(N_3_)-NH_2_ product to yield the final product (62 mg, 0.087 mmol, 34% yield). Characterize the final product by LC–MS (electrospray ionization) mass spectrometry.
20. Weigh the peptide, and dissolve in PBS at a concentration of 50 mM. Aliquot 100 μL into microcentrifuge tubes and store at −80 °C. The peptide can be stored for over a year.

## Key points

- The procedures cover a synthetic route to generate the photocaged lysine for photocaged SpyCatcher expression, the design of light-activated SpyLigation split proteins and plasmid vector design for expression in bacterial or mammalian cells.
- The light-activated SpyLigation recombinant proteins are expressed and tested in solution, tethered into engineered biomaterials by site-specifically appending biorthogonal reactive handles on photocaged SpyCatcher through a sortase-mediated reaction, and transfected into mammalian cells for intracellular protein localization/activation via photolithographic patterning.

## Related links

Key references using this protocol

Ruskowitz, E. R. et al. *Nat. Chem*. **15**, 694–704 (2023): https://doi.org/10.1038/s41557-023-01152-x

Shadish, J. A. et al. *Nat. Mater*. **18**, 1005–1014 (2019): https://doi.org/10.1038/s41563-019-0367-7

## References

[1] Shekhawat SS & Ghosh I Split-protein systems: beyond binary protein–protein interactions. Curr. Opin. Chem. Biol. 15, 789–797 (2011). 22070901 10.1016/j.cbpa.2011.10.014 PMC3237955

[2] Michnick SW, Ear PH, Manderson EN, Remy I & Stefan E Universal strategies in research and drug discovery based on protein-fragment complementation assays. Nat. Rev. Drug Discov. 6, 569–582 (2007). 17599086 10.1038/nrd2311

[3] Fink T Design of fast proteolysis-based signaling and logic circuits in mammalian cells. Nat. Chem. Biol. 15, 115–122 (2019). 30531965 10.1038/s41589-018-0181-6 PMC7069760

[4] Rihtar E Chemically inducible split protein regulators for mammalian cells. Nat. Chem. Biol. 19, 64–71 (2023). 36163385 10.1038/s41589-022-01136-x

[5] Gao Y Complex transcriptional modulation with orthogonal and inducible dCas9 regulators. Nat. Methods 13, 1043–1049 (2016). 27776111 10.1038/nmeth.4042 PMC5436902

[6] Farrar MA, Alberola-lla J & Perlmutter RM Activation of the Raf-1 kinase cascade by coumermycin-induced dimerization. Nature 383, 178–181 (1996). 8774884 10.1038/383178a0

[7] Spencer DM, Wandless TJ, Schreiber SL & Crabtree GR Controlling signal transduction with synthetic ligands. Science 262, 1019–1024 (1993). 7694365 10.1126/science.7694365

[8] Kawano F, Suzuki H, Furuya A & Sato M Engineered pairs of distinct photoswitches for optogenetic control of cellular proteins. Nat. Commun. 6, 6256 (2015). 25708714 10.1038/ncomms7256

[9] Levskaya A, Weiner OD, Lim WA & Voigt CA Spatiotemporal control of cell signalling using a light-switchable protein interaction. Nature 461, 997–1001 (2009). 19749742 10.1038/nature08446 PMC2989900

[10] Kennedy MJ Rapid blue-light–mediated induction of protein interactions in living cells. Nat. Methods 7, 973–975 (2010). 21037589 10.1038/nmeth.1524 PMC3059133

[11] Ruskowitz ER Spatiotemporal functional assembly of split protein pairs through a light-activated SpyLigation. Nat. Chem. 15, 694–704 (2023). 37069270 10.1038/s41557-023-01152-x PMC10164143

[12] Zakeri B Peptide tag forming a rapid covalent bond to a protein, through engineering a bacterial adhesin. Proc. Natl Acad. Sci. USA 109, E690–7 (2012). 22366317 10.1073/pnas.1115485109 PMC3311370

[13] Keeble AH & Howarth M Power to the protein: enhancing and combining activities using the Spy toolbox. Chem. Sci. 11, 7281–7291 (2020). 33552459 10.1039/d0sc01878c PMC7844731

[14] Keeble AH & Howarth M in Methods in Enzymology vol. 617 443–461 (Elsevier, 2019). 30784412 10.1016/bs.mie.2018.12.010

[15] Hoorens MWH & Szymanski W Reversible, spatial and temporal control over protein activity using light. Trends Biochem. Sci. 43, 567–575 (2018). 29934030 10.1016/j.tibs.2018.05.004

[16] Ruskowitz ER & DeForest CA Proteome-wide analysis of cellular response to ultraviolet light for biomaterial synthesis and modification. ACS Biomater. Sci. Eng. 5, 2111–2116 (2019). 33405713 10.1021/acsbiomaterials.9b00177

[17] Chin JW Expanding and reprogramming the genetic code. Nature 550, 53–60 (2017). 28980641 10.1038/nature24031

[18] Chen PR A facile system for encoding unnatural amino acids in mammalian cells. Angew. Chem. Int. Ed. 48, 4052–4055 (2009). 10.1002/anie.200900683 PMC2873846 19378306

[19] Young TS, Ahmad I, Yin JA & Schultz PG An enhanced system for unnatural amino acid mutagenesis in *E. coli*. J. Mol. Biol. 395, 361–374 (2010). 19852970 10.1016/j.jmb.2009.10.030

[20] Yanagisawa T Multistep engineering of Pyrrolysyl–tRNA synthetase to genetically encode N*ɛ*-(o-Azidobenzyloxycarbonyl) lysine for site-specific protein modification. Chem. Biol. 15, 1187–1197 (2008). 19022179 10.1016/j.chembiol.2008.10.004

[21] Serfling R Designer tRNAs for efficient incorporation of non-canonical amino acids by the pyrrolysine system in mammalian cells. Nucleic Acids Res. 46, 1–10 (2018). 29177436 10.1093/nar/gkx1156 PMC5758916

[22] To T-L, Zhang Q & Shu X Structure-guided design of a reversible fluorogenic reporter of protein-protein interactions: design of a reversible fluorogenic reporter of PPIs. Protein Sci. 25, 748–753 (2016). 26690964 10.1002/pro.2866 PMC4815423

[23] Hall MP Engineered luciferase reporter from a deep sea shrimp utilizing a novel imidazopyrazinone substrate. ACS Chem. Biol. 7, 1848–1857 (2012). 22894855 10.1021/cb3002478 PMC3501149

[24] Guimaraes CP Site-specific C-terminal and internal loop labeling of proteins using sortase-mediated reactions. Nat. Protoc. 8, 1787–1799 (2013). 23989673 10.1038/nprot.2013.101 PMC3943461

[25] Mao H, Hart SA, Schink A & Pollok BA Sortase-mediated protein ligation: a new method for protein engineering. J. Am. Chem. Soc. 126, 2670–2671 (2004). 14995162 10.1021/ja039915e

[26] Shadish JA, Benuska GM & DeForest CA Bioactive site-specifically modified proteins for 4D patterning of gel biomaterials. Nat. Mater. 18, 1005–1014 (2019). 31110347 10.1038/s41563-019-0367-7 PMC6706293

[27] Gawade PM, Shadish JA, Badeau BA & DeForest CA Logic-based delivery of site- specifically modified proteins from environmentally responsive hydrogel biomaterials. Adv. Mater. 31, 1902462 (2019). 10.1002/adma.201902462 PMC8296976 31265196

[28] Batalov I, Stevens KR & DeForest CA Photopatterned biomolecule immobilization to guide three-dimensional cell fate in natural protein-based hydrogels. Proc. Natl Acad. Sci. USA 118, e2014194118 (2021). 33468675 10.1073/pnas.2014194118 PMC7848611

[29] Agard NJ, Prescher JA & Bertozzi CR A strain-promoted [3 + 2] azide−alkyne cycloaddition for covalent modification of biomolecules in living systems. J. Am. Chem. Soc. 126, 15046–15047 (2004). 15547999 10.1021/ja044996f

[30] DeForest CA, Polizzotti BD & Anseth KS Sequential click reactions for synthesizing and patterning three-dimensional cell microenvironments. Nat. Mater. 8, 659–664 (2009). 19543279 10.1038/nmat2473 PMC2715445

[31] Arakawa CK, Badeau BA, Zheng Y & DeForest CA Multicellular vascularized engineered tissues through user-programmable biomaterial photodegradation. Adv. Mater. 29, 1703156 (2017). 10.1002/adma.201703156 PMC5628157 28737278

[32] Badeau BA, Comerford MP, Arakawa CK, Shadish JA & DeForest CA Engineered modular biomaterial logic gates for environmentally triggered therapeutic delivery. Nat. Chem. 10, 251–258 (2018). 29461528 10.1038/nchem.2917 PMC5822735

[33] Szymczak AL Correction of multi-gene deficiency in vivo using a single ‘self-cleaving’ 2A peptide–based retroviral vector. Nat. Biotechnol. 22, 589–594 (2004). 15064769 10.1038/nbt957

[34] Roberts PJ Rho family GTPase modification and dependence on CAAX motif-signaled posttranslational modification. J. Biol. Chem. 283, 25150–25163 (2008). 18614539 10.1074/jbc.M800882200 PMC2533093

[35] Keeble AH Approaching infinite affinity through engineering of peptide–protein interaction. Proc. Natl Acad. Sci. USA 116, 26523–26533 (2019). 31822621 10.1073/pnas.1909653116 PMC6936558

[36] Keeble AH DogCatcher allows loop-friendly protein–protein ligation. Cell Chem. Biol. 29, 339–350.e10 (2022). 34324879 10.1016/j.chembiol.2021.07.005 PMC8878318

[37] Wu X-L, Liu Y, Liu D, Sun F & Zhang W-B An intrinsically disordered peptide-peptide stapler for highly efficient protein ligation both in vivo and in vitro. J. Am. Chem. Soc. 140, 17474–17483 (2018). 30449090 10.1021/jacs.8b08250

[38] Fierer JO, Veggiani G & Howarth M SpyLigase peptide–peptide ligation polymerizes affibodies to enhance magnetic cancer cell capture. Proc. Natl Acad. Sci. USA 111, E1176–E1181 (2014). 24639550 10.1073/pnas.1315776111 PMC3977242

[39] Buldun CM, Jean JX, Bedford MR & Howarth M Snoopligase catalyzes peptide–peptide locking and enables solid-phase conjugate isolation. J. Am. Chem. Soc. 140, 3008–3018 (2018). 29402082 10.1021/jacs.7b13237

[40] Lorand L & Graham RM Transglutaminases: crosslinking enzymes with pleiotropic functions. Nat. Rev. Mol. Cell Biol. 4, 140–156 (2003). 12563291 10.1038/nrm1014

[41] Fegan A, White B, Carlson JCT & Wagner CR Chemically controlled protein assembly: techniques and applications. Chem. Rev. 110, 3315–3336 (2010). 20353181 10.1021/cr8002888

[42] Sun J & Sadelain M The quest for spatio-temporal control of CAR T cells. Cell Res. 25, 1281–1282 (2015). 26575974 10.1038/cr.2015.131 PMC4670991

[43] Barlow AD, Nicholson ML & Herbert TP Evidence for rapamycin toxicity in pancreatic β-cells and a review of the underlying molecular mechanisms. Diabetes 62, 2674–2682 (2013). 23881200 10.2337/db13-0106 PMC3717855

[44] Hartzell EJ, Terr J & Chen W Engineering a Blue Light Inducible SpyTag System (BLISS). J. Am. Chem. Soc. 143, 8572–8577 (2021). 34077186 10.1021/jacs.1c03198

[45] Wong S, Mosabbir AA & Truong K An engineered split intein for photoactivated protein trans-splicing. PLoS ONE 10, e0135965 (2015). 26317656 10.1371/journal.pone.0135965 PMC4552755

[46] Cadet J, Sage E & Douki T Ultraviolet radiation-mediated damage to cellular DNA. Mutat. Res. Mol. Mech. Mutagen. 571, 3–17 (2005). 10.1016/j.mrfmmm.2004.09.012 15748634

[47] Lawrence KP The UV/visible radiation boundary region (385–405 nm) damages skin cells and induces “dark” cyclobutane pyrimidine dimers in human skin in vivo. Sci. Rep. 8, 12722 (2018). 30143684 10.1038/s41598-018-30738-6 PMC6109054

[48] Kozmin S UVA radiation is highly mutagenic in cells that are unable to repair 7,8-dihydro-8-oxoguanine in *Saccharomyces cerevisiae*. Proc. Natl Acad. Sci. USA 102, 13538–13543 (2005). 16157879 10.1073/pnas.0504497102 PMC1224634

[49] Zhang W Optogenetic control with a photocleavable protein, PhoCl. Nat. Methods 14, 391–394 (2017). 28288123 10.1038/nmeth.4222

[50] Coin I, Beyermann M & Bienert M Solid-phase peptide synthesis: from standard procedures to the synthesis of difficult sequences. Nat. Protoc. 2, 3247–3256 (2007). 18079725 10.1038/nprot.2007.454

[51] Kawada Y, Kodama T, Miyashita K, Imanishi T & Obika S Synthesis and evaluation of novel caged DNA alkylating agents bearing 3,4-epoxypiperidine structure. Org. Biomol. Chem. 10, 5102 (2012). 22614066 10.1039/c2ob25366f

[52] Jumper J Highly accurate protein structure prediction with AlphaFold. Nature 596, 583–589 (2021). 34265844 10.1038/s41586-021-03819-2 PMC8371605

[53] Baek M Accurate prediction of protein structures and interactions using a three-track neural network. Science 373, 871–876 (2021). 34282049 10.1126/science.abj8754 PMC7612213

[54] Dolberg TB Computation-guided optimization of split protein systems. Nat. Chem. Biol. 17, 531–539 (2021). 33526893 10.1038/s41589-020-00729-8 PMC8084939

[55] Chen X, Zaro JL & Shen W-C Fusion protein linkers: property, design and functionality. Adv. Drug Deliv. Rev. 65, 1357–1369 (2013). 23026637 10.1016/j.addr.2012.09.039 PMC3726540

[56] Phillips-Jones MK, Hill LS, Atkinson J & Martin R Context effects on misreading and suppression at UAG codons in human cells. Mol. Cell. Biol. 15, 6593–6600 (1995). 8524224 10.1128/mcb.15.12.6593 PMC230912

[57] Beier H Misreading of termination codons in eukaryotes by natural nonsense suppressor tRNAs. Nucleic Acids Res. 29, 4767–4782 (2001). 11726686 10.1093/nar/29.23.4767 PMC96686

[58] Shadish JA & DeForest CA Site-selective protein modification: from functionalized proteins to functional biomaterials. Matter 2, 50–77 (2020).

[59] Stoien JD & Wang RJ Effect of near-ultraviolet and visible light on mammalian cells in culture. II. Formation of toxic photoproducts in tissue culture medium by blacklight. Proc. Natl Acad. Sci. USA 71, 3961–3965 (1974). 4530275 10.1073/pnas.71.10.3961 PMC434306

[60] DeForest CA & Tirrell DA A photoreversible protein-patterning approach for guiding stem cell fate in three-dimensional gels. Nat. Mater. 14, 523–531 (2015). 25707020 10.1038/nmat4219

